# Smart Hydrogels for Craniofacial Regeneration

**DOI:** 10.3390/cells15121054

**Published:** 2026-06-09

**Authors:** Hossein Omidian, Erma J. Gill, Umadevi Kandalam

**Affiliations:** 1 Barry and Judy Silverman College of Pharmacy, Nova Southeastern University, Fort Lauderdale, FL 33328, USA; eg1262@mynsu.nova.edu; 2 Woody L. Hunt School of Dental Medicine, Texas Tech University Health Sciences Center, El Paso, TX 79905, USA

**Keywords:** hydrogel scaffolds, craniofacial regeneration, dental stem cells, extracellular vesicles, immunomodulatory biomaterials

## Abstract

Hydrogel scaffolds have emerged as instructive microenvironments for craniofacial tissue regeneration, moving beyond passive cell carriers toward platforms that regulate cell fate, vascularization, immune remodeling, and tissue-specific architecture. This review synthesizes hydrogel-associated strategies across dental pulp, periodontal ligament, gingival, bone marrow, jawbone, endothelial, oral mucosal, induced pluripotent stem cell (iPSC), extracellular vesicle (EV), exosome, secretome, and acellular systems. The evidence indicates that craniofacial hydrogel performance is governed by reciprocal interactions among biological source, scaffold composition, matrix mechanics, spatial architecture, mineral or ionic signaling, growth factor delivery, vesicle-mediated communication, and inflammatory niche modulation. Mineralized and ion-releasing hydrogels most consistently supported osteogenesis and bone repair, whereas extracellular matrix (ECM)-mimetic, peptide, collagen, fibrin, gelatin methacryloyl (GelMA), alginate, hyaluronic acid (HA), and chitosan-based systems enabled pulp–dentin, periodontal, peri-implant, oral mucosal, and soft-tissue reconstruction. Responsive, antimicrobial, antioxidant, conductive, and immunomodulatory hydrogels further expanded the field by targeting diseased microenvironments rather than regeneration alone. Despite strong preclinical evidence, translation remains limited by heterogeneity in scaffold formulations, biological sources, analytical endpoints, defect models, and long-term functional validation. Future progress will require standardized characterization, tissue-specific design criteria, clinically relevant large-animal models, scalable cell-free technologies, and integrated assessment of regeneration, immunity, vascularization, innervation, mechanics, and safety.

## 1. Introduction

Craniofacial tissue regeneration presents a distinctive biological and engineering challenge because the target tissues are structurally heterogeneous, developmentally specialized, anatomically confined, and frequently exposed to mechanical loading, microbial challenge, and inflammatory disease. Regeneration of the pulp–dentin complex, periodontal ligament, alveolar bone, peri-implant tissues, oral mucosa, temporomandibular joint (TMJ) cartilage, and craniofacial bone require more than replacement of lost tissue volume; it requires coordinated control of cell survival, lineage commitment, matrix deposition, vascularization, immune resolution, and spatial tissue organization. Hydrogel scaffolds are particularly well positioned for this task because they can approximate ECM hydration, encapsulate or recruit regenerative cells, deliver bioactive signals, and be engineered for injectability, cross-linking, degradation, mechanical tuning, and localized release.

The synthesized evidence shows that dental and oral cell sources remain central to hydrogel-mediated craniofacial regeneration. Dental pulp-derived cells bridge endodontic and mineralized tissue repair, supporting odontoblastic, dentinogenic, pulp-like, alveolar bone, and craniofacial bone outcomes [1,2,3,4,5,6,7,8,9,10,11]. Periodontal ligament-derived cells show particularly broad regenerative potential, extending from periodontal reconstruction and alveolar bone repair to osteogenic, chondrogenic, tendon-like, neurogenic, vascular, and cementum–periodontal ligament–alveolar bone applications [12,13,14,15,16,17,18,19,20,21,22]. Gingival-derived cells and gingival-reprogrammed systems further expand the accessible oral cell repertoire, especially for periodontal, peri-implant, craniofacial bone, soft-tissue, epithelial, and cartilage-related regeneration [23,24,25,26,27,28,29,30,31,32]. These findings position hydrogel scaffolds not merely as delivery matrices, but as microenvironmental regulators whose performance depends on matching scaffold cues to the intrinsic biological identity of the delivered or recruited cell population.

Material design is equally decisive. Alginate, chitosan, collagen, fibrin, platelet-derived matrices, peptide hydrogels, GelMA, hyaluronic acid, polyethylene glycol (PEG)-based networks, mineralized composites, and semisynthetic systems were used to tune mechanics, adhesion, porosity, degradation, printability, injectability, and bioactive release [1,2,3,4,5,7,9,11,12,13,21,23,26,28,31,33,34,35,36,37,38,39,40,41,42,43,44,45,46,47,48,49,50]. Mineral phases such as hydroxyapatite, nano-hydroxyapatite, calcium phosphate, whitlockite, and magnesium-containing systems were especially prominent in craniofacial bone and alveolar repair [1,22,31,36,49,50,51,52,53]. ECM-mimetic and mechanically tunable platforms, including arginine-glycine-aspartic acid (RGD-alginate), fibronectin-functionalized PEG-hyaluronan-gelatin systems, collagen stiffness gradients, and aligned or multilayered scaffolds, demonstrate that craniofacial cell fate is shaped by biophysical and architectural cues as much as by soluble biochemical stimulation [4,18,23,32,38,43,54,55].

A major conceptual advance across the field is the transition from static scaffolds to dynamic, instructive niches. Growth factor-loaded, platelet-derived, ion-releasing, gas-releasing, antimicrobial, antioxidant, conductive, pH-responsive, reactive oxygen species (ROS)-responsive, gingipain-responsive, and EV-loaded hydrogels were used to regulate osteogenesis, angiogenesis, immunomodulation, antimicrobial defense, anti-senescence responses, and tissue patterning [14,37,52,53,56,57,58,59,60,61,62,63,64,65,66,67,68,69]. This shift is particularly important in periodontitis, peri-implantitis, diabetic inflammatory microenvironments, osteoporotic bone defects, and TMJ degeneration, where successful regeneration depends on remodeling hostile disease niches rather than merely implanting cells or osteoinductive materials [14,53,60,61,63,67,68].

Experimental models collectively support this biological complexity. Hydrogel systems were examined in two-dimensional and three-dimensional cultures, tooth slices, dentin cylinders, simulated root canals, root segments, subcutaneous implantation, calvarial and alveolar defects, periodontal and peri-implantitis models, mandibular and jaw defects, osteochondral and TMJ cartilage models, large-animal platforms, and limited clinical translation [2,3,5,6,11,15,16,17,18,20,22,24,25,26,29,32,51,54,62,65,70,71,72,73,74,75,76,77,78]. Across these models, the most consistent outcomes include enhanced viability, adhesion, migration, osteogenic differentiation, mineralization, vascularized pulp-like tissue formation, periodontal patterning, macrophage polarization, inflammatory attenuation, and organized tissue repair [1,2,3,5,9,16,20,52,53,63,65,68,69,70]. Together, these findings support a unifying thesis: hydrogel scaffolds for craniofacial regeneration are most effective when designed as biologically instructive, tissue-specific, and disease-responsive cell-regulatory systems.

## 2. Cell Source, Biological Objective, and Target Lineage

This section focuses on the biological systems used in hydrogel-associated craniofacial tissue-regeneration studies. It includes the cell population, cell-derived product, tissue origin when stated, biological objective, and intended target lineage or regenerative tissue. Scaffold composition, material chemistry, assay methods, culture or implantation time points, molecular mechanisms, and outcome measurements are excluded, except where quantitative or technical information directly defines the investigated biological cell population.

### 2.1. Dental Pulp, Apical Papilla, and Deciduous Tooth-Derived Systems

Dental pulp-derived populations represent a major biological system within the hydrogel literature. Human dental pulp stem cells (DPSCs) and related stromal populations exhibit general MSC-associated multilineage potential relevant to osteogenic and bone-regenerative targets [1,2,3,4,6,7,8,9,10,11]. The source-specific rationale for using these populations stems from their developmentally related odontogenic or dentinogenic capacity, supporting regenerative endodontics, pulp-like tissue formation, and vital pulp preservation [5,39,42,48,79].

Other localized dental sources offer related anatomical or developmental advantages. Stem cells of the apical papilla (SCAP) have been investigated for regenerative endodontic procedures and odontoblastic differentiation targets associated with the root apex [80]. Stem cells from human exfoliated deciduous teeth (SHED) serve as a clinically accessible pediatric option for craniofacial repair, while SHED-derived exosomes have been examined in periodontitis-related contexts [67,81]. DPSC-derived extracellular vesicles (EVs) or exosomes further extend this source category to cell-free signaling approaches supporting alveolar bone regeneration and periodontal healing, including interactions with Hertwig’s epithelial root sheath cells, bone marrow mesenchymal stem cells (BMSCs), and endothelial cells [58,63,65,66].

### 2.2. Periodontal Ligament and Periodontal Complex-Derived Systems

Periodontal ligament-derived populations form another central biological category bridging mineralized and soft tissues. Human periodontal ligament stem cells (PDLSCs) possess standard MSC-associated multilineage potential, including osteogenic, neurogenic, chondrogenic, and tendon differentiation under specific conditions [12,13,14,15,17,19,20,21,22,76]. Their source-specific rationale lies in targeted reconstruction of tissues anatomically associated with the periodontal complex and in periodontitis-associated inflammatory repair [12,13,14,15,17,19,20,21,22,76].

In preclinical models, transplanted rat periodontal cells contributed to alveolar bone regeneration [74], while the swine periodontal ligament fibroblast line TesPDL3 served as a model for osteoblastic and vascular lineage potential [82]. Human and canine PDLSCs have been applied to targeted alveolar bone repair [19]. To recreate complex anatomical interfaces, human PDLSC sheets have been combined with jawbone MSC sheets to generate dual-component grafts containing ligamentous and bone-like components [20]. Similarly, human dental follicle stem cells (DFSCs) use developmentally related lineage traits to guide regeneration of the cementum–periodontal ligament–alveolar bone complex [18]. Regarding cell-density parameters, PDLSCs or gingival mesenchymal stem cells (GMSCs) were encapsulated at 2 × 10^6^ cells/mL as the defined condition investigated for bone tissue engineering outcomes [75].

### 2.3. Gingival and Oral Soft-Tissue-Derived Systems

Gingival-derived systems serve as clinically accessible oral cell sources for hydrogel-associated therapies. GMSCs and gingival margin-derived progenitor cells share general MSC-associated multilineage differentiation potential across periodontal, bone, soft-tissue, myogenic, neurogenic, chondrogenic, and tendon-related targets [15,23,24,25,26,28,29,31,32]. Their source-specific rationale includes their described potential for soft-tissue re-epithelialization and epithelial sealing around dental implants [15,23,24,25,26,28,29,31,32].

Specific protocols have used 250,000 green fluorescent protein (GFP)-labeled GMSCs to evaluate cellular integration during periodontal repair [24] or anti-STRO-1 antibody sorting to define the investigated stem/progenitor population [25]. Beyond primary cells, reprogrammed systems offer alternative platforms: human gingival fibroblast-derived induced pluripotent stem cells (iPSCs) have been evaluated for periodontal differentiation propensity relative to neonatal skin-derived iPSCs, while mouse-derived gingival iPSCs have been used to generate three-dimensional osteoinductive constructs for bone regeneration [27,30].

### 2.4. Bone Marrow, Jawbone, Umbilical Cord, Endothelial, and Oral Mucosal Systems

To evaluate comparative potential or support tissue formation, hydrogels also incorporate non-dental or auxiliary mesenchymal populations. Human BMSCs, jawbone MSCs, and general human or iPSC-derived MSCs exhibit standard MSC-associated functions relevant to mineralized repair, bone engineering, and multilineage sheet assemblies [15,20,34,49,71,72,83]. Fully differentiated human osteoblastic cells provide bone-associated activity [51,52]. For injectable applications, human umbilical cord MSCs (hUCMSCs) encapsulated at 1 × 10^6^ cells/mL were investigated in relation to proliferative capacity and bone tissue engineering [50].

Beyond stem-cell osteogenesis, human umbilical vein endothelial cells (HUVECs) have been combined with DPSCs to support vascularized pulp-like tissue formation [5], while primary oral fibroblasts and keratinocytes recreate connective tissue and epithelial layers for oral mucosa tissue engineering [84]. These systems extend the section beyond stem-cell osteogenesis to vascular, epithelial, and mucosal reconstruction [5,84].

### 2.5. Cell-Free, Acellular, and Broad Biological-Regeneration Targets

Rather than delivering a defined exogenous stem-cell source, cell-free or acellular hydrogel strategies rely on endogenous host mechanisms and localized biological signaling. These platforms focus on broad tissue-repair pathways, including controlled drug delivery, biomimetic matrix properties, localized immunomodulation, cartilage-related repair, and bone defect regeneration through DNA-based hydrogels [53,59,68,77,78,85,86,87,88]. These investigations show that hydrogel physical and chemical properties can guide endogenous responses supporting pulp–dentin complex repair, vascularized soft-tissue regeneration, and structural bone healing [53,59,68,77,78,85,86,87,88].

**Figure 1:**
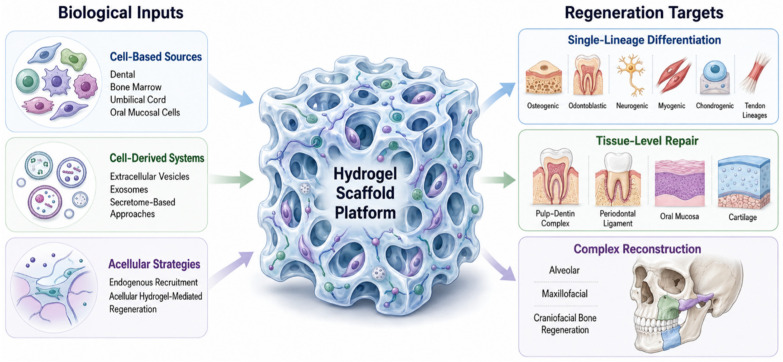
**Biological source–target logic of hydrogel scaffolds in craniofacial tissue regeneration.** Conceptual schematic showing hydrogel scaffold platforms as an interface between biological inputs and craniofacial regeneration targets. Biological inputs include stem cell-based sources, cell-derived systems, and acellular strategies. Regeneration targets are organized by increasing biological complexity, including single-lineage differentiation, tissue-level repair, and complex craniofacial reconstruction.

In summary, hydrogel-mediated craniofacial tissue regeneration spans diverse cellular and acellular configurations. General MSC-associated functions provide a shared foundation across bone marrow and oral stromal cells, whereas a source-specific rationale explains why particular cell types are selected as targets that are anatomically associated or developmentally related to their tissue of origin. Table 1 outlines these major source categories and regenerative targets, while Figure 1 illustrates the logic connecting biological inputs to lineage- and tissue-level reconstruction outcomes.

## 3. Hydrogel/Scaffold Composition and Bioactive Microenvironment

**Figure 2:**
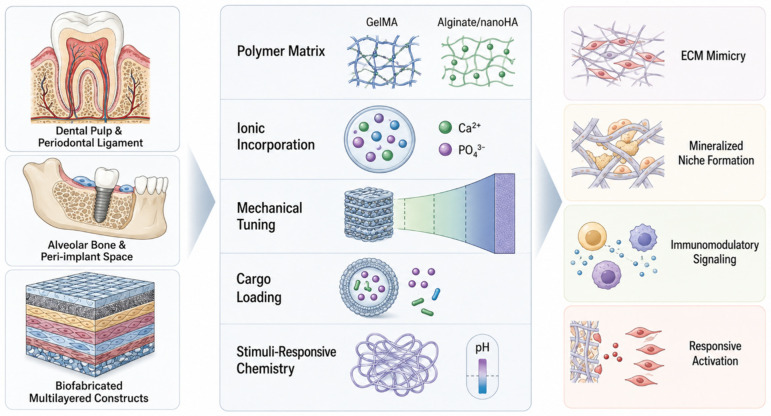
**Programmable hydrogel/scaffold microenvironments for craniofacial tissue regeneration.** The figure organizes craniofacial target environments, including dental pulp and periodontal ligament, alveolar bone and peri-implant space, and bio-fabricated multilayered constructs, in relation to major engineering levers: polymer matrix selection, ionic incorporation, mechanical tuning, cargo loading, and stimuli-responsive chemistry. These levers generate key microenvironmental functions, including ECM mimicry, mineralized niche formation, immunomodulatory signaling, and responsive activation.

This section focuses solely on the engineered hydrogel or scaffold environment used to support, encapsulate, deliver, or regulate cells or cell-derived bioactive components in craniofacial tissue-regeneration applications. Content is limited to biomaterial composition, hydrogel/scaffold architecture, cross-linking or fabrication strategy, structural and mechanical cues, incorporated bioactive agents, delivery systems, and quantitative formulation details. Figure 2 provides the conceptual framework for the section, and Table 2 summarizes the main craniofacial scaffold design strategies and representative formulation features.

### 3.1. Alginate-Based and Mineralized Craniofacial Hydrogel Systems

Alginate-based hydrogels represented one of the most widely used scaffold families across craniofacial applications, particularly when cell encapsulation, mineral incorporation, stiffness modulation, microsphere delivery, or growth factor release is required. Early alginate systems included alginate–gelatin/nano-hydroxyapatite (nanoHA) microcapsules, in which nanoHA served as a mineral ceramic phase and contributed to a rougher, more compact microcapsule surface [1]. Related alginate–gelatin bioinks were formulated as gelatin–alginate (GA), gelatin–alginate–hydroxyapatite (GAHAp), and gelatin–alginate–hydroxyapatite–PRGF (GAHAp), incorporating hydroxyapatite as a mineral cue and plasma rich in growth factors (PRGF) as a platelet-derived bioactive component [33]. Injectable three-dimensional RGD-alginate scaffolds were designed for multiple growth factor delivery, with scaffold stiffness identified as a tunable material property [23]. Alginate was also combined with hyaluronic acid to create a hydrogel scaffold for sustained human nerve growth factor (NGF) release, incorporating matrix elasticity and NGF as microenvironmental cues [13]. In another formulation, alginate was paired with GelMA, while pure alginate served as a comparator; recombinant human bone morphogenetic protein-2 (rhBMP-2) was added as a bioactive cue in the alginate/GelMA system [34]. Alginate-based hydrogels were also formulated with 30–70 weight percent (wt%) nanoHA, making nanoHA content the principal ceramic-ratio variable [51].

Several alginate systems were designed for bone, periodontal, endodontic, or craniofacial defect environments through additional mineral, adhesive, or release functions. Calcium alginate hydrogels were formulated as plastic, drug-loadable systems with variable calcium alginate concentrations and bovine serum albumin release capacity [71]. RGD-coupled alginate microspheres were loaded with silver lactate at 0.50 mg/mL, with silver ion release reported for up to 2 weeks [56]. Alginate/gelatin scaffold stiffness was tuned using 2% CaCl_2_ cross-linking: the low-stiffness hydrogel contained 2% alginate/8% gelatin and measured 11 ± 1 kPa, whereas the high-stiffness hydrogel contained 8% alginate/12% gelatin and measured 55 ± 3 kPa; the respective swelling and degradation values were 20 ± 3% and 47 ± 5% for the low-stiffness formulation, and 35 ± 2% and 18 ± 2% for the high-stiffness formulation [38]. Other alginate-based constructs included adhesive, photocrosslinkable, osteoconductive hydrogels containing MSC aggregates and hydroxyapatite microparticles [73]; RGD-alginate microencapsulation systems [16]; transforming growth factor beta 1 (TGF-β1)- or transforming growth factor beta 3 (TGF-β3)-loaded RGD-alginate microspheres [15,17]; and modified alginate hydrogels containing whitlockite microparticles, where Mg^2+^ release was identified as a material-associated cue [31]. Xeno-free alginate-fibrin hydrogel microbeads were prepared as alginate + fibrin (Alg + Fib), alginate + hPL (Alg + hPL), and alginate + fibrin + hPL (Alg + Fib + hPL) conditions, with human platelet lysate (hPL) tested at 1%, 2.5%, and 5% [47]. Additional alginate-containing systems included alginate/Matrigel/bioactive-glass microparticle composites [90], standard and three-dimensional bioprinted alginate/gelatin scaffolds [8], RGD-alginate/0.5% laponite microspheres measuring 350–450 μm and sustaining vascular endothelial growth factor (VEGF) release for 28 days [9], oxidized alginate-fibrin microbeads of 100–500 μm containing 1 × 10^6^ hUCMSCs/mL [50], injectable alginate-fibrin fibers incorporating 50 μm metformin [21], and PuraMatrix-based delivery into 6 mm tooth root segments for VEGF- or stromal cell-derived factor 1 alpha (SDF-1α)-modified DPSCs [11].

### 3.2. Chitosan and Polysaccharide-Based Injectable or Release Systems

Chitosan-containing scaffolds formed a second major group, particularly for thermoresponsive, injectable, and controlled-release craniofacial applications. Thermosensitive chitosan/β-glycerophosphate hydrogels incorporated freeze-dried platelet concentrate at 5, 10, or 15 mg/mL, providing platelet-derived factors including TGF-β1, platelet-derived growth factor-BB (PDGF-BB), and insulin-like growth factor-1 (IGF-1) [12]. A related thermosensitive quercetin-delivery system used β-glycerophosphate-chitosan/collagen hydrogels with varied chitosan/collagen ratios; the 2:1 *wt*/*wt* chitosan/collagen formulation was specifically identified, and higher chitosan content was associated with greater porosity and slower quercetin release [35]. Fibroin/chitosan oligosaccharide lactate hydrogel was used as another delivery matrix, with 250,000 GFP-labeled GMSCs delivered in 50 μL of hydrogel [24]. Injectable thermosensitive chitosan/β-glycerophosphate/hydroxyapatite hydrogels were prepared as C/GP and C/GP/HA systems, incorporating hydroxyapatite as a mineralized bioactive phase [36]. In situ injectable chitosan biguanidine/carboxymethylcellulose hydrogels were loaded with VEGF and recombinant bone morphogenetic protein-2 (BMP-2), including a CG11/BMP-2/VEGF formulation designed for sequential growth factor release [37]. Glycol chitosan-based injectable hydrogels were also engineered as single-network and double-network three-dimensional systems, with emphasis on microstructure, mechanics, rheology, and degradation [42].

Other chitosan and polysaccharide systems focused on exosome delivery, alignment, thermosensitivity, or prolonged release. A chitosan hydrogel incorporated DPSC-derived exosomes, with microRNA-1246 (miR-1246) identified as an associated exosomal component [63]. Tripolyphosphate (TPP)/chitosan beads were prepared under coagulation conditions at 4 °C with gelatin and coated with sodium alginate to form a polyelectrolyte complex film; model-drug loading efficiency exceeded 90%, with fluorescein isothiocyanate (FITC)-dextran released within 1–2 days and brilliant blue released for more than 2 months [86]. An aligned porous hydrogel was fabricated from chitosan and oxidized chondroitin sulfate using freeze-casting, establishing alignment as the principal structural cue [32]. A thermosensitive poly(N-isopropylacrylamide)-grafted chitosan (PNIPAAm-g-chitosan)/gelatin hybrid hydrogel was prepared by mixing chitosan-g-PNIPAAm with varied gelatin amounts and cross-linking with genipin; lower gelatin content was associated with higher G′ elasticity [48]. A compliant four-dimensional hydrogel was synthesized from unmodified natural polysaccharides using polyelectrolyte complexation, followed by partial coacervate compaction and dehydration, producing bondable and sequentially shape-morphing hydrogel fragments [85].

### 3.3. Peptide, Collagen, Fibrin, and Platelet-Derived Matrices

Peptide-, collagen-, fibrin-, and platelet-derived matrices were used as scaffold platforms in studies related to regenerative endodontics, periodontal regeneration, craniofacial bone repair, oral mucosa engineering, and related tissue engineering models. PuraMatrix, a self-assembling peptide hydrogel, was evaluated at 0.05–0.25% for culturing DPSCs in regenerative endodontic models [2]. PuraMatrix was also used at 0.15–0.5% as a three-dimensional culture system for PDLSCs exposed to calcium silicate-based sealer eluates, including BioRoot RCS, ProRoot ES, and mineral trioxide aggregate (MTA) Fillapex [40]. Additional PuraMatrix-based systems included combinations containing BMP-2 and/or predifferentiated gingiva-derived MSCs in a regenerating rat alveolar defect model [26]. Other peptide-based systems included a cell-adhesive, enzyme-cleavable self-assembling nanofiber hydrogel that incorporated basic fibroblast growth factor (bFGF), TGF-β1, and VEGF through heparin binding [39]; acellular injectable self-assembling peptide hydrogels designed to support vascularized pulp-like tissue formation without added external growth factors [78]; and SPG-178-Gel, an injectable self-assembling peptide scaffold evaluated for bone regeneration and three-dimensional osteogenic induction of DPSCs [6].

Collagen-based matrices included dense collagen gel scaffolds seeded with DPSCs for craniofacial bone regeneration in rat calvarial defects [70], plastically compressed collagen hydrogels seeded with SHED and used with hypoxic or fibroblast growth factor-2 (FGF-2) priming for craniofacial bone repair [81], and collagen hydrogels prepared at 3 mg/mL and 10 mg/mL for pulp–dentin complex regeneration studies [27]. In the latter study, the 3 mg/mL collagen hydrogel, Col3, had a loose structure and stiffness of 735 Pa, while the 10 mg/mL collagen hydrogel, Col10, had a condensed structure and stiffness of 8142 Pa; VEGF was loaded into Col3 at 50 ng/mL, and BMP-2 was loaded into Col10 at 50 ng/mL [4]. Collagen was also used in a polycaprolactone (PCL)/collagen/cellulose acetate electrospun scaffold system combined with cross-linked collagen hydrogels containing PDLSCs and curcumin-loaded zeolitic imidazolate framework-8 (ZIF-8) nanoparticles for periodontal ligament regeneration [14]. A separate collagen-based piezoelectric hydrogel incorporated short electrospun poly-L-lactic acid (PLLA) nanofibers and was activated by ultrasound to generate localized electrical cues in a cartilage-repair model [87]. Collagen and GelMA hydrogels were also compared as connective tissue scaffolds for oral mucosa engineering, with fibroblasts encapsulated in cross-linked hydrogels and keratinocytes cultured on the constructs [84].

Fibrin-based and platelet-derived platforms included PEGylated fibrin hydrogel for dental pulp and periodontal ligament stem-cell delivery [3], platelet-rich fibrin (PRF) prepared from six healthy volunteers and tested for effects on human dental pulp cells [91], and PRF prepared from 12 mL of patient blood by 10 min of centrifugation and placed into a root canal beneath 3 mm of grey MTA in a regenerative endodontic case report [77]. PRF scaffolds were also combined with PDLSC and jawbone mesenchymal stem cell (JBMSC) sheets in a simulated periodontal space composed of treated dentin matrix and hydroxyapatite/tricalcium phosphate frameworks [20].

### 3.4. PEG, GelMA, Hyaluronic Acid, and Semisynthetic Tunable Hydrogels

Synthetic and semisynthetic hydrogels were used in these studies to vary scaffold composition, cross-linking, cell adhesiveness, printability, EV delivery, and release behavior. A PEG-based injectable hydrogel combined polyethylene glycol diacrylate (PEGDA), hyaluronan (HA), and gelatin, with PEGSSDA cross-linker concentrations ranging from 0.5% to 8.0% *w*/*v*. In the same study, a 2% *w*/*v* PEGSSDA-HA-Gn formulation was tested with HA:Gn volume ratios from 100:0 to 25:75, and fibronectin was incorporated at 0.1, 1.0, and 10.0 μg/mL to evaluate its effect on human dental pulp stem cell (hDPSC) viability, proliferation, and spreading [43].

Gelatin methacryloyl was used as a 5% GelMA hydrogel to encapsulate hDPSCs and human umbilical vein endothelial cells (HUVECs) in tooth root segments for pulp-like tissue regeneration [5]. GelMA was also used with PEG dimethacrylate to create nanoliter-scale bioprinted gradient hydrogel arrays for screening PDLSC behavior [46], as a 5% *w*/*v* bioink for bioprinting EphrinB2-overexpressing DPSCs for alveolar bone engineering [7], as an injectable hydrogel for sustained release of cordycepin-loaded DPSC-derived exosomes in aged bone-repair models [66], and as part of porous GelMA/silk fibroin methacryloyl (SilMA) photocrosslinkable injectable hydrogels encapsulating gingiva-derived MSCs for peri-implant epithelial sealing [28]. A gelatin/poly(lactic-co-glycolic acid)-PEG-poly(lactic-co-glycolic acid) (PLGA-PEG-PLGA) nanocomposite hydrogel was loaded with TGF-β1 and had a reported pore size of approximately 202.05 μm [41]. Another gelatin-based system used microbial transglutaminase-crosslinked gelatin combined with hyaluronan or a hyaluronan–chondroitin mixture to form semi-interpenetrating hydrogels, with heteropolysaccharides retained in the hydrogels for 30 days [44].

Hyaluronic acid-based platforms included Restylane, a United States Food and Drug Administration (FDA)-approved injectable HA gel compared with Matrigel for stem cells of the apical papilla in regenerative endodontic models [80]; high-molecular-weight hyaluronan, low-molecular-weight hyaluronan, and hybrid cooperative complexes tested for osteogenic differentiation of hDPSCs [45]; and interleukin-1 receptor antagonist (IL-1ra)-loaded or unloaded HA-derived extracellular matrix (HA-sECM) used with autologous gingival margin-derived stem/progenitor cells in periodontal defects in miniature pigs [25]. Crosslinked polylysine–hyaluronic acid microspheres were developed as spherical pl-HAM carriers and combined with dental mesenchymal stem cells (DMSCs) and gelatin in one experimental condition for soft-tissue regeneration [29]. A hyaluronic acid hydrogel was also loaded with 6-bromoindirubin-3′-oxime-(BIO) encapsulated PLGA microspheres, showing an initial release phase during week 1 followed by sustained release until week 4 [89]. SHED-Cu-HA hydrogels incorporated Cu^2+^ ions and SHED-derived exosomes into hyaluronic acid for periodontal-pocket injection in periodontitis models [67]. Mussel-inspired modified hyaluronic acid adhesive hydrogels were loaded with osteoinductive DPSC-derived EVs for bone-defect repair models [65].

### 3.5. Responsive, Conductive, Ionic, and Hierarchically Organized Scaffolds

Several scaffolds incorporated advanced physical, chemical, ionic, conductive, or responsive cues to construct more actively regulated craniofacial microenvironments. A redox-active alginate/gelatin hydrogel incorporated a conductive poly(3,4-ethylenedioxythiophene)-assembled, polydopamine-mediated silk microfiber network and a hydrogen sulfide sustained-release system based on bovine serum albumin nanoparticles [57]. Decellularized, demineralized bone ECM was mixed with alginate to form ALG/ECM hydrogels, with VEGF, TGF-β3, BMP-2, parathyroid hormone-related protein (PTHrP), and vitamin D3 (VitD3) loaded into PLGA microparticles, and constructs prepared at 5 mm dimensions [72]. A biodegradable triple-layered electrospun scaffold with aligned-random-aligned architecture was functionalized with cell-specific ECM to form Bi-ECM-TLS; within this design, an aligned gelatin/PCL fibrous membrane had a water contact angle of approximately 50° and high mechanical strength [54]. A separate ion-delivery platform used monodisperse core–shell microspheres composed of a PLGA-MgO (magnesium oxide) core and an alginate hydrogel shell; the core served as the Mg^2+^ reservoir, while the shell controlled Mg^2+^ outflow at approximately 50 ppm for 2 weeks [52].

Stimuli-responsive systems included PEGPD@SDF-1 hydrogel synthesized from PEGDA, dithiothreitol, and a functional peptide module loaded with stromal cell-derived factor 1 (SDF-1); the module also contained a short antimicrobial peptide designed for gingipain-specific cleavage [60]. RGD-alginate gel was described as an injectable ROS-responsive multifunctional hydrogel, although its detailed chemical composition was not specified [61]. Acid-responsive transformable nanoparticles were assembled from a multivalent hydrophobic pH-responsive cyclodextrin host and a multivalent hydrophilic guest macromolecule; proton-triggered hydrolysis converted these nanoparticles into a localized protective hydrogel [59]. A dual-crosslinked biomimetic hydrogel contained a rigid polyhedral oligomeric silsesquioxane (POSS) core, six disulfide-linked PEG shells, and two ureidopyrimidinone (UPy) groups, combining thiol-disulfide pH-responsive chemistry with mechanically reinforcing UPy interactions [55]. A covalently immobilized BMP-2 injectable hydrogel was created by incorporating O-propargyl-tyrosine into BMP-2 and attaching BMP-2-OpgY to a methoxy PEG–PCL block copolymer through Cu(I)-catalyzed click chemistry, forming MC-BMP-2 that gelled almost immediately after injection [62].

Bone tissue regeneration remains a challenge in the clinical setting because of its complexity. To overcome these challenges, several approaches have been adopted. In one study, hierarchical, multilayered, or composite scaffolds were developed by integrating a rigid, three-dimensional PCL/nanoHA structure with a bioink containing hDPSCs, alginate, nanoHA, and collagen [79]. In another study, a trilayered nanocomposite scaffold approach was adopted to regenerate hard and soft tissues. The trilayered nanocomposite scaffold contained distinct layers of chitin-PLGA/nanoscale bioactive glass ceramic (nBGC)/cementum protein 1 as the cementum layer, chitin-PLGA/FGF-2 for the periodontal ligament layer, and chitin-PLGA/nBGC/platelet-rich plasma-derived growth factors as the alveolar bone layer [18]. A calcium phosphate cement system was compared across three distinct stem-cell populations: induced pluripotent stem cell-derived MSCs, hDPSCs, and BMSCs encapsulated in calcium phosphate cement containing hydrogel fibers, which were differentiated into the osteogenic lineage and had 62% porosity [49]. In one study, PRF scaffolds were combined with PDLSC and JBMSC sheets in a simulated periodontal space using a treated dentin matrix and hydroxyapatite/tricalcium phosphate framework, resulting in periodontal ligament-like tissue within 8 weeks [20,80]. Unmodified and alkaline phosphatase (ALP)-mineralized alginate beads co-immobilized ALP to create a mineralizing microenvironment [83]. DNA-based hydrogels were described as bulk hydrogels, microspheres, or three-dimensional printed structures incorporating phosphate ions, plasmids, oligodeoxynucleotides, tetrahedral DNA nanostructures, aptamers, loaded factors, and bound ions [88]. Additional mineral or inorganic systems included hierarchical deferoxamine@PCL nanoparticle/manganese carbonyl nanosheet/GelMA/polylactide-hydroxyapatite scaffolds with carbon monoxide (CO) and Mn^2+^ release [68], nanoHA/chitosan/gelatin three-dimensional porous scaffolds [22], cerium oxide nanoparticle- and dentin matrix protein 1 (DMP1)-loaded injectable Fmoc(fluorenylmethoxycarbonyl (Fmoc)-triphenylalanine) hydrogels [10], and injectable hydrogen-releasing Mg@PEG-PLGA hydrogels, including a 2Mg@PEG-PLGA formulation containing 2 mg Mg that transitioned in situ into a porous mechanical scaffold [53].

Some summaries reported hydrogel carriers without specifying their precise composition. These included hydrogel encapsulation in serum-based culture comparisons [92], hydrogel carriers for iPSC implantation [27], unspecified hydrogel delivery of PDLSC-derived exosomes [69], and certain reactive or thermosensitive hydrogel systems in which the functional description was provided but detailed chemistry was not specified [61,76].

## 4. Experimental Model, Controls, and Analytical Methods

This section summarizes only the experimental models, cell–material configurations, controls or comparators, quantitative technical parameters, time points, and analytical methods used to evaluate hydrogel scaffold systems for craniofacial tissue regeneration. It excludes results, mechanistic interpretation, claims of biological superiority, and conclusions about regenerative efficacy, except where markers or endpoints are named as part of the assay design. The synthesis is based exclusively on the provided summaries and previously integrated section drafts.

### 4.1. Experimental Model Hierarchy

Across the studies, hydrogel scaffolds were evaluated through a staged methodological hierarchy that included in vitro cell material testing, material characterization and release analysis, ex vivo or organotypic dental models, subcutaneous or ectopic implantation, orthotopic craniofacial defect models, and a limited number of large-animal or clinical translational models. In vitro systems commonly incorporated DPSCs, PDLSCs, GMSCs, stem cells from the apical papilla, bone marrow stromal or mesenchymal stem cells, oral fibroblasts, keratinocytes, Hertwig’s epithelial root sheath (HERS) cells, hUCMSCs, human periodontal ligament stem cells, SHED, iPSC-derived cells, and EV- or exosome-based preparations within alginate, gelatin, collagen, chitosan, hyaluronan, fibrin, GelMA, PuraMatrix, PEG-based, RGD-alginate, alginate-fibrin, peptide, or mineral-containing hydrogel systems [1,2,3,4,7,8,9,10,11,12,13,14,17,21,23,28,29,31,33,34,35,36,37,39,40,41,42,43,44,45,46,47,48,49,50,52,53,54,55,56,57,60,61,64,65,66,67,68,69,79,82,83,84,85,86,90,91,92,93,94].

Several studies used ex vivo or organotypic craniofacial models to approximate the spatial constraints of dental or mineralized tissues. These included human tooth slices, dentin cylinders, in vitro root models, simulated root canals, tooth root segments, simulated periodontal spaces, embryonic chick segmental bone defects, and human tooth-root constructs injected or filled with hydrogel systems [2,4,5,8,9,11,20,39,40,42,51]. Subcutaneous implantation in immunocompromised, nude, severe combined immunodeficient (SCID), or immunodeficient animals was used as an intermediate in vivo model for encapsulated oral stem cells, growth factor-loaded microspheres, dentin-cylinder constructs, peptide hydrogels, ALG/ECM constructs, oxidized alginate microbeads, RGD-alginate systems, BMP-2-immobilized hydrogels, and gene-modified or growth factor-modified constructs [3,4,5,9,11,13,15,16,17,23,30,39,62,72,75].

**Figure 3:**
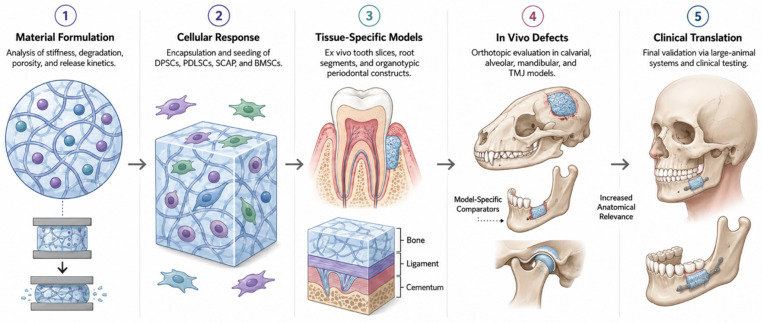
**Validation continuum for hydrogel scaffolds in craniofacial tissue regeneration.** The figure depicts a staged experimental framework moving from material formulation and characterization to cellular response testing, tissue-specific ex vivo or organotypic models, in vivo defect models, and clinical translation. The continuum emphasizes increasing anatomical relevance from physicochemical scaffold evaluation through craniofacial defects and translational testing.

Orthotopic and defect-based testing was distributed across calvarial, maxillary alveolar, mandibular, alveolar socket, jaw, femoral condyle, osteochondral, periodontal, peri-implantitis, periodontitis, temporomandibular joint osteoarthritis (TMJOA) condylar cartilage, and pulp-space models. These models were performed in mice, rats, rabbits, minipigs, beagles, and dogs, with one clinical PRF revitalization case reported in a 9-year-old patient with a necrotic immature maxillary central incisor [6,14,16,18,19,22,24,25,26,28,31,32,53,54,59,61,63,65,66,67,69,70,71,73,74,76,77,78,81,87,89]. One DNA-hydrogel review contributed a methodological synthesis of fabrication approaches, including bulk hydrogels, microspheres, and three-dimensional printing, rather than a primary experimental model [88]. The overall validation sequence is illustrated in Figure 3.

### 4.2. Controls, Comparators, and Technical Design Features

The studies used controls and comparators based on hydrogel composition, bioactive loading, stiffness, cell source, culture condition, delivery format, and defect status. Material comparisons included alginate/gelatin versus alginate/gelatin/nanoHA microcapsules [1], unloaded versus FDPC-loaded chitosan/β-glycerophosphate hydrogels containing 5, 10, or 15 mg/mL FDPC [12], GA versus GAHA versus GAHAP bioinks [33], C/GP versus C/GP/HAp hydrogels [36], Restylane versus Matrigel and stem cells from the apical papilla-only culture [80], single-network versus double-network glycol chitosan hydrogels [42], Col3 and Col10 collagen hydrogels loaded with VEGF or BMP-2 [4], and low-stiffness versus high-stiffness alginate–gelatin hydrogels cross-linked with 2% CaCl_2_ [38]. Additional formulation comparisons included variable chitosan/collagen ratios, including a 2:1 *wt*/*wt* formulation [35]; fetal bovine serum (FBS), human serum, and synthetic serum in monolayer and hydrogel cultures [92]; PEGSSDA concentrations of 0.5–8.0% *w*/*v*; 2% *w*/*v* PEGSSDA-HA-Gn hydrogels with HA:Gn ratios from 100:0 to 25:75; fibronectin at 0.1, 1.0, and 10.0 μg/mL [43]; alginate, oxidized alginate, and oxidized alginate-fibrin microbeads [50]; and hydrogel microspheres containing silver lactate, VEGF, TGF-β1, TGF-β3, SDF-1, BIO, metformin, magnesium, or exosome/EV cargos [9,15,17,21,53,56,60,65,69,89].

Cell-source comparisons included DMSCs versus human bone marrow mesenchymal stem cells (hBMMSCs) [23], PDLSCs versus DMSCs with hBMMSC controls in RGD-alginate microsphere systems [15,17], hDPSCs, hBMSCs, bone marrow-derived human induced pluripotent stem cell-derived mesenchymal stem cells (BM-hiPSC-MSCs), and foreskin-derived human induced pluripotent stem cell-derived mesenchymal stem cells (FS-hiPSC-MSCs) in calcium phosphate cement/hydrogel-fiber constructs [49], gingival induced pluripotent stem cell (G-iPSC) versus skin induced pluripotent stem cell (S-iPSC) hydrogel constructs [27], PDLSC and JBMSC sheets in PRF-based simulated periodontal constructs [20], and oral MSCs from buccal fat pad adipose tissue, periodontal ligament, and dental pulp under different serum conditions [92]. In vivo comparator designs included acellular scaffolds [70], polylactic acid (PLA)-based hydrogels [71], untreated or empty defects [5,25,26], non-RGD controls [16], blank controls or ordinary hydrogels [32], hydrogel-only and negative-control groups [22,69], MC-Cl or mixed implants in BMP-2 hydrogel testing [62], and healthy, periodontitis-only, fibroin/chitosan oligosaccharide lactate (F/COS)-only, and GMSC + F/COS groups in a rat periodontitis model [24].

Quantitative design and formulation details included 0.05–0.25% PuraMatrix [2], 0.15–0.5% PuraMatrix for three-dimensional sealer testing [40], 5% GelMA root-segment systems [5], 5% *w*/*v* bioprinted GelMA constructs [7], 3 mg/mL and 10 mg/mL collagen hydrogels loaded with 50 ng/mL VEGF or 50 ng/mL BMP-2 [4], 30–70 wt% nanoHA alginate hydrogels [51], 11 ± 1 kPa and 55 ± 3 kPa alginate–gelatin stiffness conditions [38], a gelatin/PLGA-PEG-PLGA scaffold pore size of approximately 202.05 μm [41], 1 ± 0.1 mm alginate microbeads containing 2 × 10^6^ cells/mL alginate [75], 100–500 μm microbeads containing 1 × 10^6^ hUCMSCs/mL [50], 350–450 μm RGD-alginate/0.5% laponite microspheres [9], injectable calcium phosphate cement with 62% porosity [49], 5 mm ALG/ECM constructs [72], 5 mm calvarial defects [6,16,70], 6 mm human tooth root segments [11], 7 × 1 × 1 mm maxillary alveolar defects [26], and 48 critical-sized jaw defects across 12 minipigs [22]. Other reported sample structures included *n* = 10 per group in a rat periodontitis model [24], *n* = 15 per group in a rat calvarial defect model [70], eight miniature pigs with four randomized periodontal treatments per animal [25], at least five root-segment replicates per group [5], and 250,000 GFP-labeled GMSCs delivered in 50 μL hydrogel [24].

### 4.3. Analytical Methods and Time Points

Material characterization included viscosity, injectability, gelation, swelling, degradation, rheology, stiffness or modulus, porosity, morphology, elemental analysis, water contact angle, phase transition, biodegradability, release kinetics, and scaffold architecture assessment [7,9,12,33,35,36,38,41,42,43,44,46,48,49,50,52,53,54,60,61,71,75,79,85,86,87,94]. Chemical and structural methods included scanning electron microscopy (SEM), Fourier-transform infrared spectroscopy (FTIR), proton nuclear magnetic resonance (^1^H NMR), attenuated total reflectance–Fourier-transform infrared spectroscopy (ATR-FTIR), X-ray diffraction, elemental analysis, two-photon microscopy, confocal microscopy, and three-dimensional modeling where applicable [1,2,41,44,48,54,75,82,86]. Release and delivery assays included growth factor, quercetin, silver ion, magnesium, H_2_/magnesium, VEGF, SDF-1, BIO, and model-drug release testing, including brilliant blue and FITC-dextran release from TPP/chitosan beads in 0.9% NaCl and 10 mM phosphate-buffered saline (PBS) at pH 7.4 [9,12,35,52,53,56,60,86,89].

Cellular and molecular analyses included MTT, water-soluble tetrazolium-1 (WST-1), Cell Counting Kit-8 (CCK-8), PrestoBlue, alamarBlue, colorimetric viability/proliferation assays, live/dead staining, lactate dehydrogenase (LDH) release, hemolysis testing, apoptosis or anti-apoptotic assessment, ALP activity, Alizarin Red S staining, Alcian blue staining, substrate assays, flow cytometry, quantitative reverse transcription polymerase chain reaction (qRT-PCR), quantitative polymerase chain reaction (qPCR), reverse transcription polymerase chain reaction (RT-PCR), real-time polymerase chain reaction, Western blotting, ribonucleic acid (RNA) sequencing, Milliplex cytokine quantification, enzyme-linked immunosorbent assay (ELISA), pathway-inhibition assays, and surface marker assessment [1,2,7,8,10,11,13,14,15,17,21,23,28,34,37,40,41,45,46,47,48,62,64,65,67,68,69,76,80,82,83,84,91,92]. Imaging and tissue-level analyses included soft X-ray, micro-computed tomography (micro-CT), histology, hematoxylin–eosin staining, Masson staining, immunofluorescence, immunohistochemistry, histomorphometry, morphometry, RUNX2 immunohistochemistry, horseradish peroxidase (HRP) penetration analysis, and clinical/radiographic follow-up [2,5,6,13,15,16,17,18,20,22,23,24,25,26,27,29,32,51,54,58,62,65,69,70,71,72,74,75,76,77,78].

Time points varied according to assay type and model. Short-term testing included 6, 24, and 72 h [80], 1 and 3 days [84], 5 days [91], 7 days [4,8,9,12,44,45,47,50,80], 14 days [1,4,8,27,36,43,45,47,50,80], and 21 days [1,2,4,36,41,45,47,48,50,83]. Intermediate and longer in vitro or release assessments included 28 days [1,9,72], 30 days [30,44], 4 weeks [3,11,15,17,23,75,94], week 4 release evaluation [89], and more than 2 months in selected non-cellular bead-delivery systems [86]. In vivo and clinical time points included 1 and 2 weeks [74], 2 and 8 weeks [24], 3 weeks [6], 4 and 8 weeks [5,25,26,76], 5 weeks [3,70], 8 weeks [16,20,58,75], up to 12 weeks [22], 16 weeks [25], 1 and 3 months [18], and 1-year follow-up in the clinical PRF case [77]. The craniofacial contexts, hydrogel configurations, controls, technical parameters, analytical methods, and time points are organized in Table 3.

## 5. Cellular, Molecular, and Tissue-Level Outcomes

**Figure 4:**
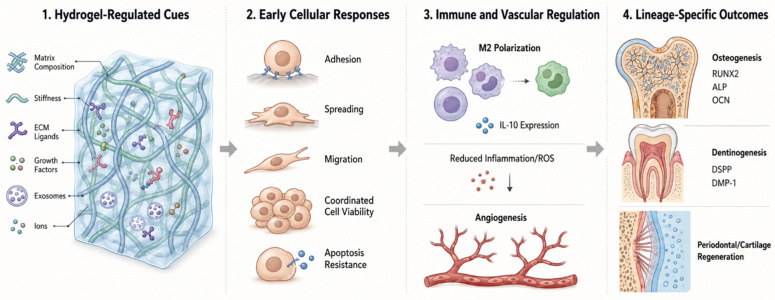
**Multiscale biological outcomes of hydrogel scaffolds in craniofacial tissue regeneration.** Hydrogel-regulated cues, including matrix composition, stiffness, ECM ligands, growth factors, exosomes, and ions, influence early cellular responses such as adhesion, spreading, migration, apoptosis resistance, and coordinated cell viability. These responses interact with immune and vascular regulation, including M2 macrophage polarization, interleukin-10 (IL-10) expression, reduced inflammation/ROS, and angiogenesis. Together, these processes support lineage-specific outcomes such as osteogenesis, dentinogenesis, periodontal/cartilage regeneration, and related craniofacial tissue repair.

Hydrogel-based and scaffold-associated systems exerted biological effects at multiple levels of craniofacial tissue regeneration, including stem-cell viability, proliferation, adhesion, spreading, migration, lineage differentiation, extracellular matrix deposition, mineralization, vascularization, immunomodulation, and tissue organization. These outcomes were reported in models of craniofacial and alveolar bone, the periodontal complex, the pulp–dentin complex, peri-implant tissues, temporomandibular joint cartilage, oral mucosa, and related soft tissues. Figure 4 provides the conceptual framework linking hydrogel-regulated cues to early cellular responses, immune–vascular regulation, and lineage-specific craniofacial outcomes.

### 5.1. Cell Viability, Proliferation, Adhesion, and Early Cell–Matrix Behavior

Hydrogel systems frequently supported early biological responses required for downstream craniofacial regeneration. hDPSCs cultured in 0.05–0.25% PuraMatrix survived and proliferated for at least 21 days, maintained healthy morphology with cytoplasmic elongations, and expressed DMP1 and dentin sialophosphoprotein (DSPP) after 21 days in tooth slices [2]. FDPC-loaded chitosan-based hydrogels significantly improved PDLSC viability after 7 days at 10 and 15 mg/mL compared with unloaded hydrogels [12]. PEGSSDA-HA-Gn hydrogels enhanced hDPSC viability over 14 days at higher HA:Gn ratios, while fibronectin significantly increased proliferation at 1.0 and 10.0 μg/mL and increased cell spreading at 0.1 μg/mL [43]. In DPSC bioinks, GAHAP showed the highest cytocompatibility, PRGF significantly enhanced adhesion and chemotaxis, and HA-containing formulations promoted the highest proliferation and osteogenic differentiation [33]. In hydrogel microarrays, PDLSC viability and spreading were composition-dependent, with both decreasing as PEG content increased [46]. Other cellular systems maintained high viability under more complex hydrogel constructs, including RGD-alginate/0.5% laponite + VEGF microspheres with hDPSC viability above 85% [9], bioprinted GelMA constructs with DPSC viability of 91.93% ± 8.38% and approximately 1.9-fold proliferation after 1 day [7], and oxidized alginate-fibrin microbeads in which live hUCMSC density at 21 days was fourfold higher than oxidized alginate and 15-fold higher than alginate alone [50].

### 5.2. Osteogenic Differentiation, Mineralization, and Craniofacial Bone Formation

Osteogenic differentiation and mineralized tissue formation were the most extensively reported outcomes in the literature. Alginate/gelatin and alginate/gelatin/nanoHA microcapsules increased hDPSC proliferation and osteogenic differentiation, with BMP-2 expression rising 1.4- and 1.7-fold at 21 days and 2.5- and fourfold at 28 days, respectively; nanoHA also upregulated osteocalcin (OCN), osteonectin, and runt-related transcription factor 2 (RUNX2) gene expression, with enhanced mineral deposition and upregulated ALP activity [1]. DPSCs in C/GP/HAp hydrogels showed adherent morphology, rapid proliferation, and high viability after 14 days, followed by significantly increased ALP activity and upregulation of RUNX2, collagen I, ALP, and OCN after 21 days [36]. VEGF/BMP-2-loaded hydrogels significantly increased DPSC proliferation and induced higher expression of ALP, collagen type I alpha 1 (COL1α1), and OCN genes and proteins, with positive mineralization and ALP activity [37]. PNIPAAm-g-chitosan/gelatin hydrogels maintained high hDPSC biocompatibility with negligible cell death, significantly increased calcium deposition after 21 days, and produced maximal ALP activity with OCN and BMP-2 expression sixfold and fourfold higher than control, respectively, in higher-gelatin formulations [48].

Several studies provided quantitative osteogenic and mineralization endpoints. In alginate-fibrin + hPL microbeads, 2.5% hPL was identified as optimal; hPL improved hPDLSC viability and release, and alginate + Fib + hPL produced ALP activity of 44.1 ± 7.61 mU/mg at 14 days, significantly exceeding alginate + Fib at 28.07 ± 5.15 mU/mg and control at 0.95 ± 0.2 mU/mg. Osteogenic genes were 3–10-fold higher than control at 7 days, and mineral deposition at 21 days was 7.5-fold and 4.3-fold higher than control for alginate + Fib + hPL and alginate + Fib, respectively [47]. In CPC hydrogel fibers, hDPSCs, bone marrow-derived human iPSC-derived MSCs, and human bone marrow mesenchymal stem cells (hBMSCs) maintained viability after injection and showed progressive mineral synthesis, with mineralization at 14 days reaching 14-fold that at day 1; mineralization did not differ significantly among these three cell types, whereas foreskin-derived human iPSC-derived MSCs showed inferior osteogenesis [49]. Osteogenic EV (Ost-EV)-loaded hydrogels increased BMP-2 expression by 2.23 ± 0.25-fold, while Ost-EVs produced higher ALP expression, greater calcium nodule formation, elevated miR-1246, reduced inflammation, accelerated revascularization, and promoted in situ mineralization [65]. Metformin-loaded alginate-fibrin fibers increased ALP and RUNX2 expression 1.4-fold, ALP activity 1.7-fold, and mineral nodule formation 2.6-fold; inhibition of the Sonic hedgehog (Shh)/glioma-associated oncogene homolog 1 (Gli1) pathway reduced osteogenic ability by 1.3- to 1.6-fold [21].

At the tissue level, these molecular and matrix findings translated into mineralized craniofacial and alveolar bone repair in multiple models. DPSC-seeded dense collagen scaffolds significantly increased bone mineral density, microarchitectural parameters, fibrous connective tissue volume, and mineralized tissue volume in rat calvarial defects compared with acellular scaffolds in *n* = 15 defects per group, with associated type I collagen, ALP, and tartrate-resistant acid phosphatase (TRAP) expression [70]. Controlled Mg^2+^ release at approximately 50 ppm for 2 weeks significantly enhanced osteoblastic activity and increased total bone volume, bone mineral density, and trabecular thickness; the Young’s modulus of newly formed bone reached approximately 96% of surrounding mature bone [52]. RGD-coupled alginate microencapsulation significantly enhanced MSC viability and osteogenic differentiation, including RUNX2, ALP, and OCN expression, and PDLSCs promoted robust mineralized repair of 5 mm calvarial defects after 8 weeks, whereas DMSCs showed lower osteogenic capacity [16]. Other bone-regenerative outcomes included complete peri-implant bone regeneration [73], significantly enhanced cleft-alveolus bone regeneration at 4 and 8 weeks [26], increased trabecular bone volume/tissue volume and trabecular thickness after ephrinB2 modification [19], significant bone and tissue repair with ordered PDL formation in aligned hydrogels [32], large normally organized vascularized bone in 48 critical-sized jaw defects across 12 minipigs [22], greater new alveolar bone formation with PDLSC-derived exosome hydrogels [69], and accelerated aged bone repair with new blood vessel formation after CY@D-exo delivery [66]. Not all formulation changes improved osteogenesis: for instance, 30 wt% nanoHA enhanced osteoblastic proliferation, osteogenic transcription factor expression, collagen deposition, trabecular bone formation, and matrix mineralization, whereas 50–70 wt% nanoHA diminished osteogenic cell response and bone tissue formation [51].

### 5.3. Pulp–Dentin Complex and Vascularized Pulp-like Tissue Formation

Hydrogel systems also supported odontoblastic differentiation, dentinogenic marker expression, reparative dentin formation, and vascularized pulp-like tissue regeneration. PEGylated fibrin supported the proliferation of dental pulp and periodontal ligament stem cells over 4 weeks, increased ALP activity after induction, upregulated osteoblast-specific genes, increased dentin-specific markers in pulp-derived stem cells, and produced collagenous matrix, mineral deposition, and vascularized soft connective tissue resembling dental pulp after 5 weeks in vivo [3]. A self-assembling peptide hydrogel promoted DPSC proliferation in three-dimensional culture and produced vascularized soft connective tissue closely resembling native dental pulp after transplantation into dentin cylinders [39]. GelMA containing hDPSCs and HUVECs generated robust pulp-like tissue that attached to the inner dentin surface, infiltrated dentinal tubules, and formed organized neovasculature, whereas acellular and empty controls produced less cellularized and poorly organized host-derived tissues [5]. Double-network glycol chitosan hydrogels facilitated hDPSC odontogenic differentiation and mineralization in vitro and supported pulp-like fibrous connective tissue in vivo [42].

Lineage-guided dentinogenic and angiogenic outcomes were also reported. In concentric collagen constructs, Col3 promoted significantly higher von Willebrand factor and cluster of differentiation 31 (CD31) expression at 7 and 14 days, whereas Col10 significantly increased DSPP, ALP, RUNX2, and collagen type I expression at 7, 14, and 21 days; the associated stiffness values were 735 Pa for Col3 and 8142 Pa for Col10, with VEGF and BMP-2 each applied at 50 ng/mL in their respective compartments [4]. Angiogenic peptide hydrogels guided neovasculature development and regenerated pulp-like soft tissue containing blood vessels, neural filaments, and an odontoblast-like layer adjacent to dentinal tubules [78]. RGD-alginate/0.5% laponite + VEGF microspheres supported abundant fibronectin and collagen I deposition at 7 days, significantly upregulated odontogenic gene expression, and promoted pulp-like tissue and microvessel regeneration after 1 month [9]. CNP/DMP1 hydrogel preserved hDPSC osteogenic/dentinogenic differentiation under inflammatory conditions, reduced pulp necrosis, promoted injured pulp repair, induced reparative dentin, and maintained pulp vitality [10]. VEGF- and SDF-1α-overexpressing DPSCs expressed significantly higher VEGF and SDF-1α levels, enhanced proliferation and vascular tube formation in vitro, and produced significantly greater regenerated pulp-like tissue length and vessel area density in vivo [11]. In a regenerative endodontic case, PRF treatment was associated at 1 year with positive cold and electric pulp-test responses, root lengthening, dentinal wall thickening, and apical closure [77].

### 5.4. Periodontal Complex, Cementum, Ligament, and Implant-Interface Outcomes

Periodontal regeneration involved coordinated outcomes in alveolar bone, cementum, periodontal ligament, epithelial interface, collagenous matrix, and inflammatory status. In vivo periodontal regeneration was robust when 250,000 GFP-labeled GMSCs were delivered in 50 μL F/COS hydrogel: in *n* = 10/group comparisons, this treatment significantly increased new bone formation at 8 weeks, reduced long junctional epithelium, and produced a better-organized PDL, whereas hydrogel alone was not significant [24]. In miniature pig periodontal defects, both IL-1ra-loaded and unloaded GMSC/HA-sECM groups showed significantly greater changes in clinical attachment level (ΔCAL), probing depth (ΔPD), and gingival recession (ΔGR), as well as higher periodontal attachment level (PAL), cementum regeneration (CR), and bone regeneration (BR), lower junctional epithelium (JE), and improved bleeding on probing (BOP) compared with negative controls [25]. PDLSC-containing scaffold-hydrogel systems promoted PDL regeneration, upregulated bFGF, hepatocyte growth factor (HGF), and TGF-β, downregulated tumor necrosis factor-alpha (TNF-α) and interleukin-6 (IL-6) and produced organized PDL structures with improved bone–cementum integration [14]. Transplanted periodontal cells directly contributed to regenerating tissues, representing approximately 2% and 1% of regenerating PDL cells at 1 week and approximately 3% at 2 weeks; in regenerating alveolar bone, labeled cells accounted for approximately 30% and 25% at 1 week, while LacZ-positive cells increased to approximately 39% at 2 weeks, and transplant sites yielded more than 40% more newly formed bone than controls [74].

Additional periodontal and peri-implant findings demonstrated organized tissue repair. Cell-specific ECM-functionalized scaffolds produced the highest bone volume/tissue volume (BV/TV) ratio, improved bone structure, optimized ligament fiber orientation, and increased blood vessel formation [54]. A trilayered scaffold favored cementogenic, fibrogenic, and osteogenic differentiation, achieved complete defect closure and healing at 1 and 3 months, and generated new cementum, fibrous PDL, and alveolar bone with well-defined trabeculae [18]. PRF-based multilayer constructs generated PDL-like tissue from PDLSC sheets, bone-like tissue from JBMSC sheets, and advanced periodontal tissue-like structures containing both functional PDL- and bone-like tissues after 8 weeks [20]. Around implants, DMSC-laden GelMA/SilMA hydrogels supported DMSC survival and proliferation, upregulated hemidesmosome-related genes, promoted M2 macrophage polarization and inhibited M1 polarization in vitro, and increased laminin subunit alpha 3 (LAMA3) and bullous pemphigoid antigen 180/collagen type XVII alpha 1 (BP180/COL17A1) expression, reduced HRP penetration, and augmented M2 macrophage infiltration in vivo [28]. pH-responsive transformable nanoparticles produced tissue-level protection through in situ hydrogel barrier formation and enabled therapeutic effects in periodontitis and arthritis models [59].

### 5.5. Immunomodulation, Inflammation Control, Angiogenesis, and Osteoimmune Regulation

Several craniofacial regenerative outcomes were associated with modulation of inflammation, macrophage phenotype, oxidative stress, vascularization, and osteoclast activity. In a three-dimensional PuraMatrix hydrogel construct, encapsulation reduced sealer-related cytotoxicity in PDLSCs, while BioRoot RCS significantly stimulated anti-inflammatory IL-10 and interleukin-4 (IL-4) release; MTA Fillapex remained strongly cytotoxic in two-dimensional culture, even at 1:4 dilution [40]. DPSC-derived exosome/chitosan (DPSC-Exo/CS) hydrogel accelerated periodontal healing, reduced lesions, suppressed inflammation, and shifted macrophages from a pro-inflammatory toward an anti-inflammatory phenotype [63]. Secretome from lipopolysaccharide (LPS)-pretreated and/or three-dimensional-cultured PDLSCs maintained good PDLSC viability, inhibited M1 macrophage polarization, promoted M1-to-M2 conversion, enhanced macrophage migration, and elevated macrophage-regulatory cytokines and growth factors [64]. SHED-Cu-HA increased hPDLSC viability and proliferation, suppressed macrophage inflammatory responses, upregulated osteogenic gene and protein expression, reduced inflammatory infiltration and bacterial infection, and promoted periodontal bone and collagen regeneration after 2 and 4 weeks [67]. PLGA-BIO-HA hydrogel inhibited periodontal inflammation, promoted bone regeneration, and induced ALP, RUNX2, and OCN expression [89].

Hydrogel-associated immune and vascular responses were also reported in bone, pulp, periodontal, and soft-tissue contexts. In diabetic periodontitis-related conditions, a hydrogel system recruited MSCs, promoted angiogenesis and osteogenesis, improved cell arrangement, increased calcium ion inflow, and enhanced bone formation [57]. RDGel encapsulation significantly enhanced the anti-apoptotic capacity of DPSCs in vitro and, in vivo, increased cartilage matrix expression, promoted subchondral bone regeneration, and favored M2 macrophage polarization during TMJ cartilage repair [61]. Polylysine–hyaluronic acid microspheres (pl-HAM) combined with DMSCs significantly promoted vascular endothelial cell migration, supported persistence of GFP-DMSCs in the regenerative area 2 weeks after surgery, increased collagen deposition, elevated CD31 expression, and recruited cluster of differentiation 44 (CD44)-, CD90-, and CD73-positive cells around microspheres [29]. Immunoregulatory bone scaffolds increased M2 macrophages, promoted VEGF-associated vascular formation, enhanced angiogenesis, weakened osteoclastogenic activity, and improved osteogenic outcomes [68]. The 2Mg@PEG-PLGA hydrogel reduced intracellular ROS, promoted M2 macrophage polarization, inhibited osteoclastogenesis, and enhanced osteogenesis in vitro and in vivo [53].

### 5.6. Non-Osteogenic Lineage Differentiation and Craniofacial Soft-Tissue Outcomes

Although osteogenesis dominated the evidence base, hydrogels also supported non-osteogenic and soft-tissue responses relevant to craniofacial regeneration. Encapsulated DMSCs and hBMMSCs developed muscle cell-like morphology and expressed high messenger RNA (mRNA) levels of myogenic differentiation 1 (MyoD), myogenic factor 5 (Myf5), and myogenin (MyoG) after 4 weeks; DMSCs showed significantly greater myogenic regenerative capacity than hBMMSCs [23]. PDLSCs and DMSCs acquired neurogenic features, including positive βIII-tubulin staining, elevated βIII-tubulin and glial fibrillary acidic protein (GFAP) expression, and dense neurogenic structures in transplanted hydrogels [13]. TGF-β1-loaded gelatin/PLGA-PEG-PLGA hydrogels improved hDPSC adhesion, viability, cytoskeletal extension, and cartilaginous matrix synthesis, and significantly increased collagen type II, SRY-box transcription factor 9 (SOX9), and aggrecan expression over 21 days [41]. TGF-β1-loaded RGD-coupled alginate microspheres supported 4-week chondrogenic differentiation, with PDLSCs, DMSCs, and control MSCs expressing collagen type II and SOX9 and producing positively stained matrices; in vivo, ectopic cartilage regenerated inside and around transplanted microspheres, and PDLSCs showed significantly greater chondrogenesis than DMSCs and control MSCs [15]. TGF-β3-loaded RGD-alginate microspheres promoted tendon-lineage differentiation, with PDLSCs, DMSCs, and hBMMSCs expressing scleraxis (SCX), decorin (DCN), tenomodulin (TNMD), and biglycan (BGN) after 4 weeks; in vivo, ectopic neo-tendon regenerated, and PDLSCs showed significantly greater tendon-regenerative capacity than DMSCs or hBMMSCs [17]. Ultrasound-activated piezoelectric hydrogels enhanced cell migration, stimulated stem cell secretion of TGF-β1, promoted chondrogenesis, increased subchondral bone formation, improved hyaline-cartilage structure, and produced mechanical properties close to those of healthy native cartilage [87]. For the oral mucosa, collagen hydrogels produced significantly higher fibroblast viability than GelMA at 24 and 72 h and supported stratified, differentiated epithelium formation, whereas GelMA did not [84].

### 5.7. Context-Dependent and Comparative Biological Responses

The biological outcomes were not uniform across all systems. GelMA increased MSC viability but reduced osteogenic differentiation in alginate/GelMA hydrogels, although rhBMP-2 restored osteogenic activity; after 2 weeks, alginate/GelMA-encapsulated MSCs retained cluster of differentiation 146 (CD146) expression, whereas cells in pure alginate did not [34]. Quercetin β-glycerophosphate chitosan/collagen hydrogels with a 2:1 chitosan/collagen ratio promoted hPDLSC growth in a dose-dependent manner [35]. Synthetic serum reduced morphological and surface antigen variation across human buccal fat pad adipose-derived stem cells (hBFP-ADSCs), hPDLSCs, and hDPSCs; osteoblastic differentiation of hPDLSCs and hBFP-ADSCs increased in synthetic serum and encapsulation models, whereas αvβ3 and β1 integrin expression was higher in FBS than in synthetic serum [92]. High-stiffness alginate–gelatin hydrogels produced significantly stronger osteogenic activity than low-stiffness hydrogels, with reported stiffness values of 55 ± 3 kPa and 11 ± 1 kPa, swelling of 35 ± 2% and 20 ± 3%, and degradation of 18 ± 2% and 47 ± 5%, respectively, where these values were linked to differential DPSC outcomes [38]. ALG/ECM hydrogels alone produced dense tissue and mineralized bone formation, host tissue invasion, and vascularization after 28 days; exogenous growth factors did not further enhance bone formation, ultraviolet (UV) irradiation reduced bone formation, and BMP-2 and VitD3 restored osteogenesis [72]. G-iPSC hydrogel constructs formed more mineralized structures and exhibited stronger periodontal-marker staining than S-iPSC constructs [27]. Overall, the evidence indicates that cellular and tissue-level outcomes varied by cell source, biological additive, EV or growth factor use, hydrogel stiffness, matrix composition, ion content, and disease microenvironment, while remaining directly measurable through viability, proliferation, lineage-marker expression, ECM deposition, mineralization, vascularization, inflammation control, and regenerated tissue organization.

Table 4 summarizes the major craniofacial outcome patterns and representative cellular, molecular, matrix, and tissue-level endpoints without duplicating the full narrative synthesis.

## 6. Mechanistic Interpretation and Regenerative Significance

**Figure 5:**
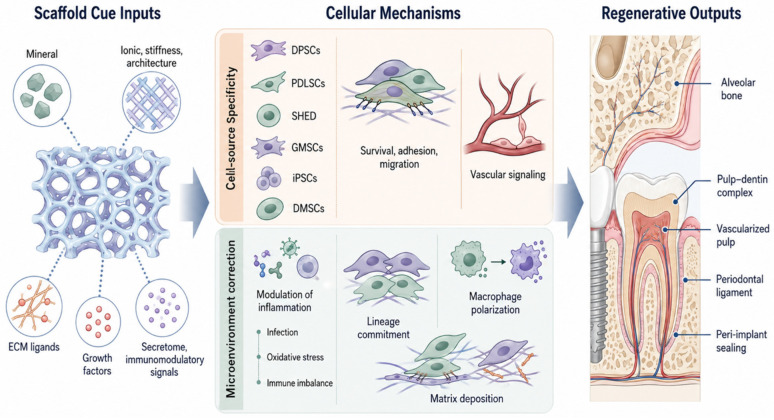
**Cell-instructive hydrogel niches in craniofacial tissue regeneration.** The figure illustrates how scaffold cue inputs—including mineral, ionic, stiffness, architectural, ECM-ligand, growth-factor, secretome, and immunomodulatory signals—regulate cellular mechanisms such as survival, adhesion, migration, lineage commitment, vascular signaling, macrophage polarization, matrix deposition, and correction of inflammatory, infectious, oxidative, or immune-imbalanced microenvironments. These mechanisms converge on craniofacial regenerative outputs, including alveolar bone, pulp–dentin complex, vascularized pulp, periodontal ligament, and peri-implant sealing, with cell-source specificity represented as a key determinant of response.

Hydrogel scaffolds for craniofacial tissue regeneration serve as cell-instructive microenvironments that regulate cell survival, adhesion, proliferation, migration, lineage commitment, paracrine signaling, immune responses, angiogenesis, and matrix deposition. Across the studies, regenerative outcomes were shaped by reciprocal interactions between scaffold-derived cues and cell-specific biological potential. These mechanisms are summarized conceptually in Figure 5, and the major scaffold-mediated design patterns are organized in Table 5.

### 6.1. Mineralized and Ionic Hydrogel Niches

Mineralized hydrogels functioned as instructive niches for osteogenic and odontogenic commitment. NanoHA-containing alginate/gelatin microcapsules enhanced hDPSC proliferation, calcium deposition, ALP activity, and osteogenic gene expression, supporting the role of mineral phases in directing dental stem cells toward bone-forming phenotypes [1]. This effect was formulation dependent: 30 wt% nanoHA enhanced osteoblastic proliferation, collagen deposition, trabecular bone formation, and mineralization, whereas 50–70 wt% nanoHA reduced osteogenic responses [51]. Related systems, including chitosan/β-glycerophosphate/hydroxyapatite (C/GP/HAp) hydrogels, calcium alginate hydrogels, nanoHA/chitosan/gelatin scaffolds, CPC with hydrogel fibers, and ALP-mineralized alginate beads, similarly preserved cell viability while promoting osteogenic differentiation or mineralized matrix formation [22,36,49,71,83].

Ion-releasing hydrogels added a second regulatory layer. Controlled Mg^2+^ release at approximately 50 ppm for 2 weeks enhanced osteoblastic activity and restored the Young’s modulus of regenerated bone to approximately 96% of surrounding mature bone [52]. Whitlockite microparticles promoted DMSC osteogenesis more effectively than hydroxyapatite microparticles, with mitogen-activated protein kinase (MAPK) activation and suppression of osteoclastic activity through Mg^2+^ release and osteoprotegerin secretion [31]. Mg/H_2_-releasing hydrogels reduced oxidative stress, promoted M2 macrophage polarization, inhibited inhibitor of κB/nuclear factor kappa B (IκB/NF-κB) signaling, enhanced osteogenesis, and suppressed osteoclastogenesis [53]. These findings indicate that mineral and ionic cues are not merely structural additives; they regulate osteogenesis, mineralization, inflammatory balance, and biomechanical recovery when concentration, release profile, and matrix context are appropriately tuned.

### 6.2. Matrix Mechanics, Architecture, and ECM-Mediated Cell Interaction

Hydrogel stiffness, elasticity, porosity, spatial organization, and ECM-like ligand presentation were central regulators of craniofacial cell behavior. High-stiffness alginate–gelatin hydrogels, 55 ± 3 kPa compared with 11 ± 1 kPa low-stiffness gels, enhanced DPSC osteogenesis while preserving adhesion and proliferation [38]. In collagen systems, matrix mechanics were paired with growth factors to direct lineage patterning: loose Col3 hydrogels at 735 Pa combined with VEGF favored vascular differentiation, whereas condensed Col10 hydrogels at 8142 Pa combined with BMP-2 enhanced odontogenic/osteogenic differentiation [4]. Scaffold stiffness also regulated DMSC myogenic differentiation in RGD-modified alginate hydrogels, while GelMA improved MSC viability but reduced stiffness-dependent osteogenesis unless rhBMP-2 was added [23,34].

Architecture and ECM composition further shaped cell–matrix interaction. Aligned porous hydrogels promoted more ordered PDL formation than ordinary hydrogels [32]. Cell-specific ECM-functionalized triple-layered scaffolds improved bone volume, ligament fiber orientation, and vascular formation, while an aligned gelatin/PCL membrane with a water contact angle of approximately 50° supported favorable periodontal cell interaction [54]. Fibronectin supplementation in PEGDA-HA-gelatin hydrogels increased hDPSC proliferation at 1.0 and 10.0 μg/mL and enhanced cell spreading at 0.1 μg/mL [43]. Heteropolysaccharide-containing gelatin hydrogels retained HA/chondroitin components for 30 days, improved stiffness, and accelerated calcified matrix formation [44]. Mechanistically, HA cooperative complexes acted through CD44-associated Yes-associated protein/transcriptional coactivator with PDZ-binding motif (YAP/TAZ) activation, and POSS-PEG-UPy hydrogels promoted PDLSC osteogenesis through ten-eleven translocation methylcytosine dioxygenase 2 (TET2)/histone deacetylase 1 (HDAC1)/E-cadherin/β-catenin signaling [45,55]. Together, these studies show that craniofacial hydrogels regulate cell fate through both physical mechanics and biochemical matrix recognition.

### 6.3. Bioactive Signaling Depots and Lineage-Specific Instruction

Many hydrogels operated as localized signaling reservoirs that controlled cell proliferation, chemotaxis, differentiation, and tissue formation. FDPC-loaded chitosan/β-glycerophosphate hydrogels released TGF-β1 and PDGF-BB for 2 weeks, and 10–15 mg/mL FDPC increased PDLSC viability [12]. PRGF enhanced DPSC adhesion and chemotaxis in HAp-containing bioinks [33]. Dual VEGF/BMP-2 hydrogels enhanced DPSC proliferation and mineralizing differentiation, while peptide nanofiber hydrogels delivering bFGF, TGF-β1, and VEGF promoted DPSC proliferation and vascularized pulp-like tissue formation [37,39]. PDGF-BB-overexpressing hPDLSCs in thermosensitive hydrogels increased proliferation, osteogenic gene expression, and alveolar bone growth, whereas covalently immobilized BMP-2 hydrogels induced mineralized calcium deposition in human periodontal ligament stem cell-loaded constructs [62,76].

Platelet-derived and small-molecule systems also provided instructive cues. PRF stimulated dental pulp cell (DPC) proliferation, osteoprotegerin (OPG) expression, and ALP activity, while 2.5% hPL in alginate-fibrin hydrogels improved hPDLSC viability, release, ALP activity, osteogenic gene expression, and mineral deposition [47,91]. BIO delivered from PLGA microspheres within HA hydrogel produced early release followed by sustained delivery to week 4, suppressing inflammation while promoting bone regeneration and osteogenic marker expression [89]. Metformin-loaded alginate-fibrin fibers enhanced hPDLSC osteogenesis through Shh/Gli1 signaling, increasing ALP activity 1.7-fold and mineral nodule formation 2.6-fold; pathway inhibition reduced osteogenic ability [21]. These findings show that hydrogel scaffolds can regulate craniofacial regeneration by controlling when, where, and how cells receive trophic, osteogenic, angiogenic, or lineage-specific signals.

### 6.4. Immunomodulatory, Antioxidant, Antimicrobial, and Disease-Responsive Microenvironments

The mechanistic evidence also shows that craniofacial regeneration depends on correcting inflammatory, oxidative, infectious, or disease-associated microenvironments. Silver lactate-loaded RGD-alginate microspheres preserved stem-cell viability at 0.50 mg/mL while providing antimicrobial activity and sustained silver ion release for up to 2 weeks, linking infection control with osteogenic cell delivery in peri-implantitis [56]. In diabetic periodontitis, redox-active H_2_S-releasing conductive hydrogels reduced oxidative and inflammatory stress, recruited MSCs, promoted angiogenesis and osteogenesis, enhanced calcium ion inflow, amplified PDLSC autophagy, and modulated macrophage polarization [57]. ROS-responsive DPSC/RDGel inhibited the p38/p53 mitochondrial apoptotic pathway, improved oxidative stress conditions, promoted M2 macrophage polarization, and enhanced cartilage and subchondral bone regeneration [61].

Other responsive or immunoregulatory systems similarly shifted the local environment toward repair. Gingipain-responsive PEGPD@SDF-1 hydrogels released antimicrobial peptide in response to *P. gingivalis*, promoted PDLSC proliferation, migration, and osteogenic differentiation, reduced inflammation, recruited CD90^+^/cluster of differentiation 34-negative (CD34^−^) stromal cells, and induced osteogenesis [60]. Curcumin-loaded ZIF-8 (CURZIF-8)-containing collagen hydrogel systems supported PDLSC viability and migration, enhanced anti-inflammatory and antioxidative activity, increased pro-healing factors, and reduced TNF-α and IL-6 [14]. SHED-Cu-HA hydrogels enhanced periodontal ligament stem-cell viability and proliferation while suppressing macrophage inflammatory responses through the IL-6/Janus kinase 2/signal transducer and activator of transcription 3 (JAK2/STAT3) pathway [67]. Osteoimmunity-regulating hierarchical scaffolds released CO and Mn^2+^ to increase M2 macrophages, promote VEGF-associated vascular formation, inhibit osteoclast activity, and synergize with HA osteoinductivity [68]. These findings show that hydrogel scaffolds can improve regeneration by transforming hostile craniofacial disease environments into pro-reparative niches.

### 6.5. Vesicle, Secretome, and Gene-Enhanced Hydrogel Systems

EV, exosome, secretome, and gene-enhanced hydrogel systems extended scaffold function from structural support to paracrine and molecular regulation. DPSC-derived exosomes in chitosan hydrogel accelerated periodontal healing by suppressing inflammation and converting macrophages from pro-inflammatory to anti-inflammatory phenotypes, with miR-1246 implicated in this response [63]. LPS-pretreated PDLSC secretome generated in three-dimensional SupraGel inhibited M1 macrophage polarization, promoted M2 transition, and enhanced macrophage migration [64]. DPSC-derived EV-loaded hydrogels promoted HERS osteogenic differentiation through TGF-β1-associated Smad, MAPK, and extracellular signal-regulated kinase (ERK) phosphorylation and increased alveolar bone formation [58]. Ost-EV-loaded adhesive hydrogels increased BMP-2 expression, enhanced ALP expression and calcium nodule formation compared with normal EVs, and promoted inflammation resolution, revascularization, and in situ mineralization [65]. PDLSC exosomes delivered by hydrogel promoted BMSC proliferation, osteogenic differentiation, and early alveolar bone formation [69].

Gene-enhanced approaches further showed that molecularly modified cells can improve scaffold-mediated regeneration. EphrinB2 overexpression enhanced osteogenic transcription and mineral deposition in periodontal and dental pulp stem cells, supporting alveolar bone formation when combined with PuraMatrix or GelMA-based constructs [7,19]. VEGF- and SDF-1α-overexpressing DPSChs in PuraMatrix enhanced proliferation, vascular tube formation, regenerated pulp-like tissue length, and vessel density within 6 mm root segments after 4 weeks [11]. CY@D-exos released from GelMA hydrogels rejuvenated senescent BMSCs and endothelial cells by activating nuclear factor erythroid 2-related factor 2 (NRF2) signaling and maintaining heterochromatin stability, linking anti-senescence mechanisms with osteogenesis and angiogenesis in aged bone repair [66]. These systems demonstrate that hydrogel scaffolds can regulate craniofacial repair through vesicle cargo, secreted factors, genetic modification, and recipient-cell signaling.

### 6.6. Vascularized Pulp, Periodontal Patterning, and Cell-Source Specificity

Hydrogels also regulated complex craniofacial tissue organization by supporting vascularization, pulp-like tissue formation, periodontal patterning, and cell-source-specific regeneration. PuraMatrix supported DPSC survival and proliferation for at least 21 days at 0.05–0.25% and permitted odontoblastic differentiation in tooth slices [2]. Restylane supported SCAP survival over 72 h and odontoblastic differentiation by 7–14 days [80]. PEGylated fibrin supported dental stem-cell proliferation over 4 weeks and vascularized pulp-like connective tissue after 5 weeks [3]. GelMA containing hDPSCs and HUVECs generated vascularized pulp-like tissue attached to dentin and infiltrating dentinal tubules [5]. Acellular angiogenic peptide hydrogels guided host neovasculature, neural filament, and odontoblast-like layer formation without exogenous growth factors [78]. RGD-alginate/0.5% laponite microspheres, 350–450 μm in size, sustained VEGF release for 28 days, maintained hDPSC viability above 85%, and promoted pulp-like tissue and microvessel formation after 1 month [9].

For periodontal and craniofacial tissue patterning, trilayered nanocomposite scaffolds provided spatially distinct cues for cementogenic, fibrogenic, and osteogenic differentiation [18]. PDLSCs, DMSCs, DPSCs, SHED, GMSCs, iPSCs, and JBMSCs showed distinct regenerative propensities across scaffold systems. PDLSCs outperformed DMSCs or hBMMSCs in several RGD-alginate contexts, including chondrogenic, tendon-like, and calvarial bone regeneration [15,16,17]. Transplanted periodontal cells directly contributed approximately 30–39% of cells in regenerating alveolar bone and produced more than 40% greater new bone than controls [74]. G-iPSCs showed stronger periodontal differentiation than S-iPSCs, consistent with retained somatic memory [27]. PDLSC sheets tended toward PDL-like tissues, whereas JBMSC sheets predominantly generated bone-like tissues within PRF-based constructs [20]. FGF-2 priming of SHED in compressed collagen hydrogels enhanced proliferation and osteogenic differentiation more strongly than hypoxia and accelerated intramembranous bone formation [81]. These findings indicate that hydrogel scaffolds regulate, but do not erase, intrinsic cell identity; effective craniofacial regeneration depends on matching scaffold cues to the biological potential of the delivered or recruited cell population.

Overall, the evidence supports a coherent mechanistic interpretation: hydrogel scaffolds for craniofacial tissue regeneration regulate cell fate through reciprocal interactions between cells and engineered microenvironments. Mineral phases, ionic release, matrix stiffness, elasticity, ECM ligands, growth factors, platelet-derived signals, vesicles, secretomes, immune cues, antimicrobial systems, vascular signals, and cell-source-specific properties collectively determine survival, migration, lineage commitment, vascular organization, matrix deposition, mineralization, and tissue-specific repair. Table 5 summarizes these scaffold-mediated mechanisms and the representative craniofacial contexts in which they operate.

## 7. Limitations and Future Perspectives

Despite substantial progress, the evidence base remains constrained by heterogeneity in cell sources, scaffold chemistries, formulation parameters, bioactive cargos, model systems, and analytical endpoints. Dental pulp, periodontal ligament, gingival, bone marrow, jawbone, umbilical cord, endothelial, oral mucosal, iPSC, EV, exosome, secretome, and acellular systems were all represented, but direct comparisons across these sources were limited and often confounded by differences in scaffold composition, culture conditions, defect models, and outcome measures [15,16,17,20,23,27,49,92]. Future studies should prioritize head-to-head comparisons using standardized hydrogel platforms and matched biological endpoints to determine when tissue-specific oral cell sources are superior to broader mesenchymal or cell-free approaches.

A second limitation is the variable depth of scaffold characterization. Many studies reported detailed composition, stiffness, swelling, degradation, porosity, release kinetics, and fabrication parameters, whereas others described hydrogel carriers without sufficient chemical or architectural definition [27,61,69,76,92]. Because small changes in matrix stiffness, mineral content, cross-linking density, ligand presentation, and release kinetics can redirect cell behavior, future work should establish minimal reporting standards for craniofacial hydrogels, including mechanical properties under physiologic conditions, degradation profiles, bioactive cargo release, sterilization effects, injectability or handling, batch reproducibility, and post-implantation material fate [4,9,31,38,43,50,52].

The field also remains dominated by short- to intermediate-term preclinical endpoints. Although in vitro assays, subcutaneous implantation, calvarial defects, periodontal defects, root-segment models, and small-animal disease models provide useful mechanistic information, they do not fully reproduce the vascular, immune, microbial, biomechanical, and anatomical complexity of human craniofacial tissues [2,5,6,11,15,16,17,20,24,25,26,29,32,51,62,65,69,70,71,72,74,75,76]. Large-animal models, including minipig and canine systems, and the limited clinical evidence available are therefore especially valuable but remain underrepresented [22,77,78]. Future studies should integrate clinically scaled defects, load-bearing environments, oral microbial exposure, immune competence, and longer follow-up periods to evaluate not only new tissue formation but also functional maturation, integration, durability, innervation, vascular stability, and safety.

Another translational barrier is the limited integration of regenerative outcomes with the control of the disease microenvironment. Several responsive or immunomodulatory systems demonstrated that antimicrobial, antioxidant, anti-inflammatory, macrophage-polarizing, anti-osteoclastogenic, and angiogenic regulation can improve regeneration in hostile craniofacial niches [14,53,56,57,60,61,63,64,67,68]. However, these approaches are still fragmented across different disease models and bioactive systems. Future hydrogel design should explicitly integrate tissue-forming cues with immune-, vascular-, neural-, and microbiome-aware functions, particularly for periodontitis, peri-implantitis, diabetic periodontal defects, osteoporotic bone defects, and TMJ degeneration.

Cell-free and molecularly enhanced hydrogels represent a particularly promising direction. EV-, exosome-, secretome-, and gene-enhanced systems regulated osteogenesis, angiogenesis, macrophage phenotype, inflammation, anti-senescence responses, and vascularized pulp-like tissue formation [7,11,19,58,63,64,65,66,69]. These strategies may reduce some risks associated with live-cell transplantation while preserving paracrine and instructive regenerative activity. However, translation will require rigorous control of vesicle source, cargo variability, potency assays, storage stability, dosing, release kinetics, biodistribution, immunogenicity, and manufacturing scalability.

Finally, future craniofacial hydrogel research should move from demonstrating regenerative potential toward defining design rules. The most informative studies will connect scaffold parameters—mechanics, architecture, degradation, ionic signaling, adhesive ligands, spatial patterning, bioactive release, and immune responsiveness—to specific cell behaviors and tissue outcomes. Emerging opportunities include multilayered and gradient scaffolds for periodontal interfaces, vascularized and innervated pulp–dentin constructs, injectable immunoregulatory hydrogels for inflammatory defects, biofabricated patient-specific scaffolds, and acellular vesicle-based systems for scalable clinical translation [5,7,9,11,18,22,54,65,66,67,68,69,78,79]. The next phase of the field will depend on whether hydrogel scaffolds can be engineered not only to regenerate tissue, but also to recreate the dynamic craniofacial niche required for durable, functional, and clinically predictable repair.

## 8. Conclusions

Hydrogel scaffolds have redefined craniofacial tissue engineering by transforming regenerative platforms from passive matrices into biologically instructive microenvironments. Across dental, periodontal, bone, mucosal, peri-implant, and cartilage-related applications, their regenerative value arises from the coordinated regulation of cell survival, lineage specification, matrix deposition, vascularization, immune remodeling, antimicrobial defense, and tissue architecture. The strongest evidence supports hydrogel systems that integrate tissue-specific biological sources with tunable mechanics, ECM-mimetic cues, mineral or ionic signaling, controlled bioactive delivery, and disease-responsive functionality. Yet the field now requires a decisive shift from proof-of-concept regeneration toward standardized, scalable, mechanistically defined, and clinically validated scaffold systems. Properly engineered, hydrogel scaffolds can become next-generation craniofacial niche therapeutics capable of guiding complex tissue repair with precision, adaptability, and translational relevance.

## Figures and Tables

**Table 1:** Hydrogel-associated biological sources and target lineages in craniofacial tissue regeneration.

Hydrogel-Associated Biological Source	Source Identity/Defining Technical Detail	Craniofacial Regenerative Target or Lineage	Pattern Shown Within Hydrogel-Based Craniofacial Regeneration	Representative References
Dental pulp-derived cellular systems	Human dental pulp stem/stromal cells, DPSCs, and dental pulp cells derived from dental tissues	Regenerative endodontics, odontoblastic/odontogenic and dentinogenic differentiation, pulp-like tissue regeneration, pulp–dentin complex repair, dental tissue regeneration, craniofacial bone, and alveolar bone regeneration	Dental pulp-derived cells bridge endodontic soft-tissue regeneration with mineralized craniofacial bone, dentin, and pulp–dentin repair	[1,2,3,4,6,8,9,10,11]
Dental pulp-derived cell-free products	DPSC-derived EVs or exosomes	Alveolar bone regeneration, periodontal healing, extensive bone-defect repair, aged bone regeneration, and modulation of target cells such as Hertwig’s epithelial root sheath cells, BMSCs, and endothelial cells	DPSC-derived vesicle systems represent a cell-free extension of dental pulp-based hydrogel regeneration	[58,63,65,66]
Apical papilla and deciduous tooth-derived systems	Stem cells of the apical papilla; SHED; SHED-derived exosomes	Regenerative endodontics, odontoblastic differentiation, craniofacial bone repair, and periodontitis-related regeneration	Developmental dental sources are used more selectively for endodontic, craniofacial bone, and periodontal inflammatory repair contexts	[67,80,81]
Periodontal ligament and periodontal complex-derived systems	PDLSCs, periodontal ligament cells, rat periodontal cells, TesPDL3 swine periodontal ligament fibroblasts, PDLSC sheets, and dental follicle stem cells; one study used 2 × 10^6^ stem cells/mL when PDLSCs or dental mesenchymal stem cells were delivered	Periodontal tissue regeneration, periodontal ligament regeneration, alveolar and maxillofacial bone repair, osteogenesis, neurogenesis, chondrogenesis, tendon differentiation, vascular lineage potential, and cementum–periodontal ligament–alveolar bone regeneration	Periodontal-derived sources show the widest lineage range and are central to both periodontal reconstruction and craniofacial hard-tissue repair	[12,13,15,17,18,19,20,22,74,75,82]
Gingival and oral soft-tissue-derived systems	GMSCs, gingiva-derived MSCs, gingival margin-derived stem/progenitor cells, gingival fibroblast-derived iPSCs, and mouse gingival fibroblast-derived iPSCs; one study used 250,000 GFP-labeled GMSCs	Periodontal regeneration, alveolar bone repair, craniofacial or maxillofacial bone regeneration, peri-implant regeneration, epithelial sealing, re-epithelialization, soft-tissue repair, myogenic, neurogenic, chondrogenic, tendon-related, and cartilage-related objectives	Gingival sources function as accessible oral MSC reservoirs, particularly where periodontal, peri-implant, soft-tissue, and craniofacial bone repair intersect	[23,24,25,26,27,28,29,30,31,32]
Bone marrow, jawbone, umbilical cord, and general MSC systems	BMSCs, bone marrow stromal cells, jawbone MSCs, iPSC-derived MSCs, general human MSCs, human osteoblastic cells, and human umbilical cord MSCs; one study used 1 × 10^6^ hUCMSCs/mL	Osteogenic differentiation, bone engineering, craniofacial or oral bone repair, chondrogenesis, tendon regeneration, periodontal reconstruction, and bone substitute development	These systems mainly serve as comparator, auxiliary, or broader mesenchymal platforms for evaluating hydrogel-based craniofacial regeneration	[15,20,34,49,50,51,71,72,83]
Endothelial and oral mucosal tissue-forming systems	Human umbilical vein endothelial cells; human primary oral fibroblasts and keratinocytes isolated from gingival biopsies	Vascularized pulp-like tissue formation and oral mucosa tissue engineering	These sources expand the biological scope beyond stem-cell osteogenesis to vascular, epithelial, connective tissue, and oral mucosal reconstruction	[5,84]
Acellular, cell-free, or non-cell-specific hydrogel targets	Acellular hydrogel-mediated systems, secretome-based approaches, endogenous recruitment strategies, DNA-based hydrogel concepts, and non-cell-specific regenerative platforms	Pulp–dentin complex regeneration, vascularized pulp-like soft-tissue regeneration, localized tissue protection in periodontitis, cartilage healing, large-scale bone-defect repair, osteoporotic bone-defect regeneration, and broad tissue-regeneration applications	These studies show that hydrogel-based craniofacial regeneration can proceed through acellular, endogenous, or cell-derived strategies rather than direct transplantation of a named stem-cell source	[53,59,68,77,78,85,86,87,88]

**Table 2:** Craniofacial hydrogel/scaffold microenvironment strategies and representative formulation features.

Craniofacial Application Context	Dominant Scaffold Strategy	Key Formulation or Manufacturing Data Retained in This Section	Main Microenvironmental Cue Represented	Representative References
Dental pulp, endodontic, and pulp–dentin regeneration	Injectable peptide, collagen, HA, GelMA, alginate, and microsphere-based systems used in tooth slices, root segments, pulp spaces, or pulp-capping settings	PuraMatrix 0.05–0.25%; PuraMatrix 0.15–0.5%; Restylane HA gel; 5% GelMA; collagen at 3 and 10 mg/mL; VEGF or BMP-2 at 50 ng/mL; RGD-alginate/0.5% Lap microspheres of 350–450 μm with 28-day VEGF release; 6 mm tooth root segments; cerium oxide nanoparticle (CNP)/DMP1-loaded Fmoc-triphenylalanine hydrogel	ECM mimicry, HA injectability, collagen stiffness, VEGF/BMP-2 delivery, EV/exosome loading, dentin-associated molecular cues, and mineral-associated pulp-capping design	[2,4,5,9,10,11,39,40,80]
Periodontal ligament and periodontal defect regeneration	Chitosan, HA, alginate, collagen, PRF, aligned, and compartmentalized scaffolds for periodontal-pocket delivery, periodontal ligament (PDL) support, or periodontal-layer organization	Freeze-dried platelet concentrate (FDPC) at 5, 10, or 15 mg/mL; 2:1 *wt*/*wt* chitosan/collagen; BIO-loaded PLGA microspheres in HA with week-1 burst and sustained release to week 4; freeze-cast chitosan/oxidized chondroitin sulfate aligned hydrogel; trilayered chitin-PLGA/nBGC or FGF-2 compartments	Platelet-derived factors, quercetin delivery, BIO release, scaffold alignment, PRF matrix cues, and cementum–PDL–alveolar bone compartmentalization	[12,18,20,29,32,35,89]
Alveolar and craniofacial bone regeneration	Mineralized alginate, chitosan, calcium phosphate, collagen, magnesium, whitlockite, hydroxyapatite, and nanoHA scaffolds	alginate/nanoHA at 30–70 wt%; alginate/gelatin stiffness range of 11 ± 1 to 55 ± 3 kPa; calcium phosphate cement with 62% porosity; alginate-whitlockite microparticle hydrogel; nanoHA/chitosan/gelatin porous scaffolds; 2Mg@PEG-PLGA with 2 mg Mg; oxidized alginate-fibrin microbeads of 100–500 μm with 1 × 10^6^ cells/mL	Mineral ceramic phases, hydroxyapatite/nanoHA, Mg^2+^ or H_2_ release, whitlockite microparticles, calcium phosphate structure, and mineralizing alginate bead systems	[22,31,36,38,49,50,51,53]
Peri-implant, antimicrobial, inflammatory, and immunomodulatory craniofacial microenvironments	Responsive or antimicrobial hydrogels designed for infection-prone, oxidative, or inflammatory oral microenvironments	Silver lactate at 0.50 mg/mL in RGD-alginate microspheres; alginate/gelatin hydrogel with poly(3,4-ethylenedioxythiophene) (PEDOT)/polydopamine silk microfiber network and bovine serum albumin (BSA) nanoparticle-based H_2_S release; PEGPD@SDF-1 with gingipain-cleavable peptide module; SHED-Cu-HA containing Cu^2+^ and SHED-derived exosomes; ROS-responsive RDGel	Silver ion release, H_2_S delivery, conductive networks, gingipain-responsive antimicrobial peptide release, Cu^2+^ incorporation, exosome loading, and ROS responsiveness	[56,57,60,61,67]
Biofabricated, architecturally organized, and spatially patterned constructs	Bioprinted, electrospun, adhesive, multilayered, and hierarchical scaffolds designed to reproduce craniofacial spatial organization	Triple-layer electrospun aligned-random-aligned scaffold; aligned gelatin/PCL membrane with approximately 50° water contact angle; rigid PCL/nanoHA structure integrated with alginate/nanoHA/collagen bioink; 5% *w*/*v* GelMA bioink; GelMA/PEG dimethacrylate nanoliter-scale gradient arrays; trilayered scaffold for cementum, PDL, and alveolar bone	Structural alignment, printability, spatial gradients, mineralized polymer frameworks, adhesive or compartmentalized craniofacial scaffold architecture	[7,8,18,46,54,79]
Synthetic, semisynthetic, and mechanically tunable hydrogel platforms	PEG, GelMA, HA, gelatin, SilMA, PLGA-PEG-PLGA, and POSS/PEG/UPy networks for tuning mechanics, adhesiveness, and degradation	PEGSSDA 0.5–8.0% *w*/*v*; 2% *w*/*v* PEGSSDA-HA-Gn; HA:Gn ratios of 100:0 to 25:75; fibronectin at 0.1, 1.0, and 10.0 μg/mL; gelatin/PLGA-PEG-PLGA pore size of approximately 202.05 μm; heteropolysaccharide retention for 30 days; POSS core with six disulfide-linked PEG shells and two UPy groups	Cross-linking density, matrix stiffness, fibronectin-mediated adhesion, HA/GelMA hybridization, pH-responsive chemistry, mechanical reinforcement, and tunable degradation	[28,41,43,44,45,46,55]
Bioactive delivery and stimuli-responsive craniofacial scaffold systems	Hydrogel or scaffold platforms delivering growth factors, ions, vesicles, gases, drugs, or nucleic acid-based cues	PLGA-MgO/alginate microspheres with Mg^2+^ outflow of approximately 50 ppm for 2 weeks; MC-BMP-2 gelation almost immediately after injection; hPL at 1%, 2.5%, and 5%; metformin at 50 μm; DNA hydrogels formed as bulk hydrogels, microspheres, or three-dimensional printed structures	Growth factor immobilization or release, Mg^2+^ delivery, platelet-derived cues, small-molecule loading, and nucleic acid-based phosphate/plasmid/oligodeoxynucleotide/tetrahedral DNA nanostructure (TDN)/aptamer cues	[21,47,52,62,83,88]

**Table 3:** Craniofacial experimental contexts, controls, and analytical methods used to evaluate hydrogel scaffold systems.

Craniofacial Testing Context	Hydrogel Scaffold and Cell/Material Configuration	Experimental Model, Controls, and Key Technhical Details	Analytical Methods and Time Points	References
Pulp–dentin and regenerative endodontic systems	PuraMatrix, Restylane, Matrigel comparator systems, GelMA, collagen hydrogels, PEGylated fibrin, peptide nanofiber hydrogels, RGD-alginate/Lap microspheres, C/GP and C/GP/HAp, chitosan biguanidine/carboxymethylcellulose, 9-fluorenylmethoxycarbonyl (Fmoc)-triphenylalanine composites, and PuraMatrix-delivered gene-modified DPSCs or stem cells from the apical papilla (SCAP)	In vitro DPSC or SCAP culture; 0.05–0.25% PuraMatrix; 0.15–0.5% PuraMatrix sealer testing; 5% GelMA root segments; tooth-slice models; dentin cylinders; simulated root canals; 3 mg/mL and 10 mg/mL collagen hydrogels with 50 ng/mL VEGF or BMP-2; RGD-alginate/Lap microspheres of 350–450 μm with 0.5% laponite; 6 mm human tooth root segments; SCID/nude mouse implantation; controls included SCAP-only, Matrigel, acellular GelMA, empty root segments, alternative stiffness conditions, and growth factor- or gene-modified groups	WST-1, MTT, CCK-8, LDH, hemolysis, live/dead staining, confocal microscopy, SEM, qRT-PCR, real-time polymerase chain reaction, Western blotting, ALP, Alizarin Red S staining, histology, immunostaining, and micro-CT; time points included 6, 24, and 72 h; 7, 14, 21, and 28 days; 1 month; and 4–8 weeks	[2,3,4,5,9,10,11,36,37,39,40,41,42,80]
Periodontal and alveolar regeneration models	F/COS hydrogel, HA-sECM, PuraMatrix, RGD-alginate, chitosan/oxidized chondroitin sulfate, BIO-loaded HA, SHED-Cu-HA, DPSC-Exo/CS hydrogel, trilayered chitin-PLGA scaffold, PCL/collagen/cellulose acetate scaffold with collagen hydrogel, PDLSC/GMSC constructs, and PDGF-BB-overexpressing human periodontal ligament stem cell thermosensitive hydrogels	Rat and mouse experimental periodontitis; rat periodontal defects; rat PDL injury; rabbit maxillary periodontal defects; miniature-pig periodontal defects; rat peri-implantitis; beagle critical-sized alveolar defects; controls included healthy, periodontitis-only, F/COS-only, GMSC + F/COS, untreated defects, scaling and root planing, scaffold-only, ordinary hydrogels, blank controls, and material-only groups; quantitative design included *n* = 10 per group, eight miniature pigs with four randomized treatments per animal, and 250,000 GFP-labeled GMSCs in 50 μL hydrogel	Micro-CT, histology, hematoxylin–eosin (HE) staining, Masson staining, immunohistochemistry, immunofluorescence, ELISA, clinical/radiographic parameters, periodontal measurements, Gene Ontology enrichment, RT-PCR, Western blotting, and HRP penetration analysis; time points included 1–2 weeks, 2 and 8 weeks, 4 and 8 weeks, 16 weeks, and 1 and 3 months	[14,18,19,20,24,25,26,28,32,54,59,63,64,67,74,76,82,89]
Craniofacial bone, calvarial, mandibular, and jawbone models	Dense collagen scaffolds, calcium alginate hydrogels, alginate/gelatin/nanoHA, alginate-based nanoHA hydrogels, alginate/GelMA, ALG/ECM, RGD-alginate, alginate–whitlockite, PCL/nanoHA-alginate/nanoHA/collagen bioink hybrids, nanoHA/chitosan/gelatin porous scaffolds, SPG-178-Gel, EV- or exosome-loaded adhesive hydrogels, calcium phosphate cement (CPC) with hydrogel fibers, Mg@PEG-PLGA hydrogel, and deferoxamine (DFO)@PCL/MnCO/GelMA/PLA-HA hierarchical scaffolds	Rat critical-size calvarial defects; murine calvarial defects; athymic nude rat maxillary alveolar defects; rabbit mandibular bone defects; rabbit subcutaneous implantation; rat alveolar defects; rat femoral condyle defects; osteoporotic bone-defect models; aged-animal bone-injury models; minipig critical-sized jaw defects; controls included acellular scaffolds, PLA, untreated defects, hydrogel-only, material-only, negative controls, and alternative stem-cell or scaffold groups; technical details included two 5 mm calvarial defects, 7 × 1 × 1 mm maxillary defects, 5-mm constructs, 62% porosity CPC, and 48 jaw defects across 12 minipigs	Micro-CT, soft X-ray, histology, histomorphometry, HE staining, Masson staining, RUNX2 immunohistochemistry, SEM, X-ray diffraction, qRT-PCR, Western blotting, ALP, Alizarin Red S staining, RNA sequencing, ROS assays, macrophage-polarization assays, and release testing; time points included 1 and 14 days, 3 weeks, 4 and 8 weeks, 5 weeks, 8 weeks, up to 12 weeks, and 28 days	[1,6,16,22,26,31,34,49,51,52,53,58,62,65,66,68,69,70,71,72,79,81,83]
Subcutaneous and ectopic implantation systems	RGD-alginate microspheres, alginate/hyaluronic acid hydrogels, PEGylated fibrin, peptide nanofibers, ALG/ECM, oxidized alginate microbeads, TGF-β1- or TGF-β3-loaded microspheres, BMP-2-immobilized hydrogels, polylysine–hyaluronic acid microspheres, thermoresponsive hydrogel cell chambers, and dentin-cylinder constructs	Subcutaneous implantation in immunocompromised, nude, SCID, or immunodeficient mice or rats; constructs included dental pulp, periodontal ligament, gingival, bone marrow, STRO-1-enriched, SHED, iPSC-derived, or hBMMSC comparator cells; controls included alternative oral MSC sources, hBMMSCs, acellular/material controls, non-RGD controls, MC-Cl or mixed implants, and growth factor variants; technical details included 5 mm ALG/ECM constructs, 1 ± 0.1 mm microbeads with 2 × 10^6^ cells/mL, and ectopic implantation afterh in vitro induction	Histology, immunofluorescence, immunohistochemistry, histomorphometry, micro-CT, SEM, X-ray diffraction, qPCR, matrix staining, migration assays, and cell-recruitment assays; time points included 2 weeks, 28 days, 4 weeks, 5 weeks, and 8 weeks	[3,4,5,9,11,13,15,16,17,23,29,30,39,62,72,75]
Material optimization, fabrication, and release-focused testing	PEGDA/HA/gelatin hydrogels, alginate–gelatin stiffness hydrogels, quercetin β-glycerophosphate-chitosan/collagen hydrogels, FDPC-loaded chitosan/β-glycerophosphate, TPP/chitosan beads, four-dimensional polysaccharide hydrogels, microfluidic PLGA-MgO/alginate microspheres, pH-responsive transformable nanoparticles, DNA-based hydrogel fabrication approaches, oxidized alginate systems, and PNIPAAm-g-chitosan/gelatin hydrogels	In vitro or materials-focused testing of viscosity, swelling, degradation, stiffness, porosity, microstructure, release, injectability, transformation mechanics, bonding, and shape change; variables included 5–15 mg/mL FDPC, 2:1 chitosan/collagen ratio, 0.5–8.0% *w*/*v* PEGSSDA, HA:Gn ratios from 100:0 to 25:75, fibronectin at 0.1–10.0 μg/mL, low/high stiffness of 11 ± 1 and 55 ± 3 kPa, alginate microbead oxidation level, and 4 °C TPP/chitosan bead preparation	Viscosity testing from 25 to 37 °C, rheology, FTIR, SEM, water contact angle, swelling/degradation assays, release kinetics, phase transition, degradation in PBS at 37 °C, cross-sectional analysis, 0.9% NaCl and 10 mM PBS release testing, and fabrication-method review; time points included 1 h, 1 day, 1–2 weeks, 28 days, week 4, and >2 months for selected bead-release systems	[12,35,38,43,48,50,52,85,86,88,94]
Peri-implant, oral mucosal, and soft-tissue models	Adhesive photocrosslinkable alginate hydrogel, silver lactate RGD-alginate microspheres, porous GelMA/SilMA hydrogels, crosslinked polylysine–hyaluronic acid microspheres, collagen or GelMA oral mucosa constructs, and PCL/collagen/cellulose acetate scaffold–collagen hydrogel systems	Rat peri-implantitis or early implant-placement models; titanium-disc antimicrobial testing; suspension bacterial-load testing; six-well insert oral mucosa constructs; human primary oral fibroblasts and keratinocytes isolated from gingival biopsies; L-929/DMSC recruitment systems; subcutaneous soft-tissue models; controls included collagen versus GelMA, scaffold-only/hydrogel-only groups, and alternative scaffold–hydrogel combinations	PrestoBlue, antimicrobial assays against *Aggregatibacter actinomycetemcomitans*, titanium-disc assays, silver ion release for up to 2 weeks, migration assays, immunofluorescence, ELISA, histology, HRP penetration, macrophage-polarization assays, hemidesmosome-related gene/protein analysis, and tissue staining; time points included 1 and 3 days and 2 weeks	[14,28,29,56,73,84]
TMJ, cartilage, tendon, and osteochondral models	ROS-responsive RDGel with DPSCs, gelatin/PLGA-PEG-PLGA-TGF-β1 hydrogels, TGF-β1- or TGF-β3-loaded RGD-alginate microspheres, and ultrasound-activated piezoelectric collagen/PLLA hydrogels	In vitro chondrogenic or tendon induction; DPSCs, PDLSCs, DMSCs, and hBMMSC comparator cells; subcutaneous ectopic cartilage or tendon testing; TMJOA condylar cartilage-defect evaluation; rabbit osteochondral critical-size defects under targeted ultrasound activation; technical details included a scaffold pore size of approximately 202.05 μm and 4-week induction systems	Viability assays, phalloidin/4′,6-diamidino-2-phenylindole (DAPI), Alcian blue staining, real-time RT-PCR, qPCR, matrix staining, histochemical staining, immunofluorescence, histology, immunohistochemistry, histomorphometry, cartilage matrix assessment, subchondral bone assessment, and macrophage-polarization endpoints; time points included 21 days and 4 weeks	[15,17,41,61,87]
Large-animal and clinical translational models	Acellular angiogenic self-assembling peptide hydrogels and PRF-based endodontic revitalization	Canine orthotopic pulpminspace implantation after pulpectomy; clinical PRF case in a 9-year-old patient with a necrotic immature maxillary central incisor; clinical protocol included canal irrigation with 20 mL 5.25% sodium hypochlorite and 10 mL 0.2% chlorhexidine, 21-day triple-antibiotic paste dressing, PRF prepared from 12 mL blood centrifuged for 10 min, and 3 mm grey MTA placement	Orthotopic pulp-space tissue assessment, clinical testing, radiographic follow-up, cold/electric pulp testing, and 1-year follow-up	[77,78]

**Table 4:** Representative cellular, molecular, and tissue-level outcome patterns of hydrogel scaffolds for craniofacial tissue regeneration.

Craniofacial Regenerative Target	Dominant Biological Outcome Pattern	Key Endpoints and Representative Findings	Representative References
Craniofacial and alveolar bone regeneration	Hydrogel systems most consistently promoted osteogenic differentiation, mineralized matrix deposition, and bone repair.	Increased BMP-2, RUNX2, ALP, OCN, osteonectin, osteopontin (OPN), collagen type I (COL-I)/COL1α1, Sp7 transcription factor (SP7), bone gamma-carboxyglutamate protein (BGLAP), sclerostin (SOST), and DMP1; increased ALP activity, calcium deposition, mineralized nodules, trabecular bone formation, bone mineral density, BV/TV, trabecular thickness, and new bone volume. Representative quantitative findings include BMP-2 increases of 1.4- and 1.7-fold at 21 days and 2.5- and fourfold at 28 days; ALP activity of 44.1 ± 7.61 mU/mg in alginate + Fib + hPL; 14-fold mineralization increase at 14 days versus day 1; and 1.7-fold ALP activity with 2.6-fold mineral nodule formation after metformin delivery.	[1,16,21,22,47,49,52,65,70]
Periodontal complex regeneration	Periodontal repair involved coordinated regeneration of alveolar bone, PDL, cementum, collagenous matrix, epithelial interface, and inflammatory control rather than bone formation alone.	Outcomes included reduced long junctional epithelium, improved PDL organization, higher BV/TV, optimized ligament fiber orientation, new cementum, fibrous PDL, alveolar bone with trabeculae, improved bone–cementum integration, and enhanced epithelial-interface markers. Representative findings include significant new bone formation after delivery of 250,000 GFP-labeled GMSCs in 50 μL hydrogel; higher ΔCAL, ΔPD, ΔGR, PAL, CR, and BR with lower JE; and increased OPN, RUNX2, COL-I, LAMA3, and BP180/COL17A1.	[14,18,20,24,25,28,32,54]
Dental pulp–dentin complex regeneration	Hydrogels supported odontoblastic differentiation, dentinogenic marker expression, reparative dentin formation, and vascularized pulp-like tissue development.	Endpoints included DMP1, DSPP, matrix extracellular phosphoglycoprotein (MEPE), ALP, RUNX2, collagen type I, CD31, von Willebrand factor, VEGF, SDF-1α, fibronectin, and collagen I. Tissue-level findings included dentinal tubule infiltration, odontoblast-like layers adjacent to dentinal tubules, vascularized pulp-like connective tissue, microvessel formation, reparative dentin, reduced pulp necrosis, and preserved pulp vitality. Representative quantitative findings include hDPSC viability above 85% in RGD-alginate/0.5% laponite + VEGF microspheres and significantly higher VEGF/SDF-1α expression with increased regenerated pulp-like tissue length and vessel area density.	[2,3,4,5,9,10,11,78,80]
Craniofacial immunomodulation and vascularization	Several regenerative outcomes were associated with inflammatory suppression, macrophage phenotype shifts, oxidative-stress reduction, angiogenesis, and reduced osteoclastogenic activity.	Findings included increased IL-10 and IL-4 release, reduced cytotoxicity, suppression of inflammatory responses, M1-to-M2 macrophage conversion, enhanced macrophage migration, reduced ROS, reduced osteoclastogenesis, increased VEGF-associated vascular formation, neovasculature, revascularization, CD31 expression, and improved tissue integration. These effects were especially relevant in periodontal, pulp, peri-implant, bone, and inflammatory defect models.	[29,40,53,63,64,65,67,68]
TMJ cartilage, chondrogenic, tendon-like, myogenic, neurogenic, and oral mucosal outcomes	A smaller but important subset of systems directed non-osteogenic differentiation or supported craniofacial soft-tissue repair.	Chondrogenic outcomes included collagen type II, SOX9, aggrecan, cartilage-like matrix, hyaline-cartilage structure, and subchondral bone formation. Tendon-like outcomes included SCX, DCN, TNMD, and BGN expression with ectopic neo-tendon formation. Other outcomes included MyoD, Myf5, and MyoG expression; βIII-tubulin and GFAP expression; neurogenic structures; and stratified differentiated oral epithelium on collagen hydrogels.	[13,15,17,23,41,84,87]

**Table 5:** Hydrogel scaffold–mediated mechanisms regulating cell fate in craniofacial tissue regeneration.

Hydrogel Scaffold Mechanism	Craniofacial Regenerative Context	Key Cell-Regulatory Data Retained for Comparison	Mechanistic Pattern Revealed	Representative References
Mineralized and ion-releasing hydrogel niches	Alveolar, jaw, calvarial, craniofacial bone, and mineralized dental tissue regeneration	NanoHA-, HA-, CPC-, whitlockite-, Mg-, and ALP-mineralized systems promoted osteogenic differentiation, mineral deposition, and bone formation. NanoHA at 30 wt% enhanced osteogenesis, whereas 50–70 wt% reduced responses; Mg^2+^ release at approximately 50 ppm for 2 weeks enhanced osteoblastic activity and restored regenerated bone modulus to approximately 96% of mature bone.	Mineral and ionic cues act as osteogenic regulators, but concentration, release kinetics, and scaffold compatibility determine benefit.	[1,22,31,36,49,51,52,83]
Matrix stiffness, elasticity, and architecture	Bone, pulp–dentin complex, PDL, myogenic differentiation, and peri-implant tissue organization	High-stiffness alginate–gelatin hydrogels, 55 ± 3 kPa versus 11 ± 1 kPa, enhanced DPSC osteogenesis; Col3 at 735 Pa with VEGF favored vascular differentiation, whereas Col10 at 8142 Pa with BMP-2 favored odontogenic/osteogenic differentiation; aligned hydrogels promoted ordered PDL formation.	Hydrogel mechanics and spatial architecture guide lineage selection and tissue patterning rather than only providing structural support.	[4,23,32,34,38,54,55]
Adhesive and ECM-mimetic cell–matrix cues	Periodontal, pulpal, peri-implant, soft-tissue, and mineralized tissue regeneration	RGD, PRGF, fibronectin, collagen, HA/chondroitin, laponite, and cell-specific ECM improved adhesion, spreading, chemotaxis, migration, matrix deposition, and organized tissue formation. Fibronectin increased proliferation at 1.0 and 10.0 μg/mL and spreading at 0.1 μg/mL.	Craniofacial hydrogel performance depends on instructive ECM recognition, not only bulk material biocompatibility.	[9,29,33,43,44,45,54]
Growth factor, platelet-derived, and small-molecule signaling depots	Periodontal regeneration, pulp regeneration, dentin repair, tendon/chondrogenic differentiation, and craniofacial bone repair	FDPC released TGF-β1 and PDGF-BB for 2 weeks, with 10–15 mg/mL FDPC increasing PDLSC viability; 2.5% hPL improved hPDLSC osteogenic activity; metformin increased ALP activity 1.7-fold and mineral nodules 2.6-fold through Shh/Gli1 signaling.	Hydrogels act as localized signaling reservoirs that regulate cell fate through temporally controlled trophic, osteogenic, angiogenic, and lineage-specific cues.	[12,15,17,21,37,39,47,62,76,89,91]
Immunomodulatory, antioxidant, antimicrobial, and disease-responsive hydrogels	Periodontitis, peri-implantitis, diabetic periodontitis, TMJ cartilage repair, osteoporotic bone defects, and inflammatory craniofacial repair	Silver lactate at 0.50 mg/mL preserved cell viability while providing antimicrobial activity; RDGel inhibited p38/p53 mitochondrial apoptosis; Mg/H_2_ systems inhibited IκB/NF-κB signaling; gingipain-responsive and copper/HA-based systems reduced inflammation while supporting stem-cell function.	Regeneration requires remodeling diseased inflammatory, oxidative, or infectious niches, not only inducing differentiation.	[14,53,56,57,60,61,63,64,67,68]
EV-, exosome-, secretome-, and gene-enhanced hydrogel systems	Alveolar bone, periodontal repair, aged bone regeneration, and vascularized pulp regeneration	DPSC-, PDLSC-, and SHED-derived vesicles or secretomes regulated osteogenesis, angiogenesis, macrophage phenotype, inflammation, and anti-senescence effects. EphrinB2-, VEGF-, and SDF-1α-modified cells enhanced mineral deposition, vascular tube formation, regenerated pulp-like tissue length, and vessel density.	Hydrogel scaffolds can deliver molecular and paracrine instructions, bridging cell-based and cell-free craniofacial regeneration.	[7,11,19,58,63,64,65,66,69]
Vascularized pulp and pulp–dentin patterning	Regenerative endodontics, pulp-like tissue formation, odontoblastic differentiation, and revascularization	PuraMatrix supported DPSC survival for ≥21 days at 0.05–0.25%; Restylane supported SCAP survival over 72 h and odontoblastic differentiation by 7–14 days; RGD-alginate/0.5% laponite microspheres, 350–450 μm, sustained VEGF release for 28 days and maintained hDPSC viability above 85%.	Functional endodontic regeneration depends on combining stem-cell survival with vascular, odontoblastic, and dentin-associated organization.	[2,3,5,9,11,78,80]
Cell-source specificity and lineage memory within hydrogel scaffolds	PDL, alveolar bone, tendon/cartilage-like tissues, craniofacial bone, and multilayer periodontal constructs	PDLSCs, DMSCs, DPSCs, SHED, GMSCs, G-iPSCs, S-iPSCs, JBMSCs, and periodontal cells showed distinct lineage propensities. Periodontal cells contributed approximately 30–39% of regenerating alveolar bone cells and produced >40% more new bone than controls; PDLSCs outperformed DMSCs or hBMMSCs in several RGD-alginate systems.	Scaffold design must be matched to cell-source biology; hydrogels guide but do not erase intrinsic regenerative identity.	[15,16,17,20,27,74,81]

## Data Availability

No new data was created or analyzed in this study. Data sharing is not applicable.

## Abbreviations

**2D**—two-dimensional; **3D**—three-dimensional; **4D**—four-dimensional; **ALP**—alkaline phosphatase; **ATR-FTIR**—attenuated total reflectance–Fourier-transform infrared spectroscopy; **bFGF**—basic fibroblast growth factor; **BGLAP**—bone gamma-carboxyglutamate protein; **BGN**—biglycan; **BIO**—6-bromoindirubin-3′-oxime; **BM-hiPSC-MSCs**—bone marrow-derived human induced pluripotent stem cell-derived mesenchymal stem cells; **BMP-2**—bone morphogenetic protein-2; **BMSCs**—bone marrow mesenchymal stem cells; **BOP**—bleeding on probing; **BP180/COL17A1**—bullous pemphigoid antigen 180/collagen type XVII alpha 1; **BR**—bone regeneration; **BSA**—bovine serum albumin; **BV/TV**—bone volume/tissue volume; βIII-tubulin—beta III tubulin; **C/GP**—chitosan/β-glycerophosphate; **C/GP/HAp**—chitosan/β-glycerophosphate/hydroxyapatite; **CCK-8**—Cell Counting Kit-8; **CD31**—cluster of differentiation 31; **CD34**—cluster of differentiation 34; **CD44**—cluster of differentiation 44; **CD73**—cluster of differentiation 73; **CD90**—cluster of differentiation 90; **CD146**—cluster of differentiation 146; **CNP**—cerium oxide nanoparticle; **CO**—carbon monoxide; **COL-I**—collagen type I; **COL1α1**—collagen type I alpha 1; **CPC**—calcium phosphate cement; **CR**—cementum regeneration; **CURZIF-8**—curcumin-loaded zeolitic imidazolate framework-8; **CY@D-exos**—cordycepin-loaded dental pulp stem cell-derived exosomes; **DAPI**—4′,6-diamidino-2-phenylindole; **DCN**—decorin; **DFO**—deferoxamine; DFSCs—dental follicle stem cells; **DMP1**—dentin matrix protein 1; **DMSCs**—dental mesenchymal stem cells; **DNA**—deoxyribonucleic acid; **DPC**—dental pulp cell; **DPSC-Exo/CS**—dental pulp stem cell-derived exosome/chitosan hydrogel; **DPSCs**—dental pulp stem cells; **DSPP**—dentin sialophosphoprotein; **ECM**—extracellular matrix; **ELISA**—enzyme-linked immunosorbent assay; **ERK**—extracellular signal-regulated kinase; **EV**—extracellular vesicle; **F/COS**—fibroin/chitosan oligosaccharide lactate; **FBS**—fetal bovine serum; **FDPC**—freeze-dried platelet concentrate; **FDA**—United States Food and Drug Administration; **FGF-2**—fibroblast growth factor-2; **FITC**—fluorescein isothiocyanate; **Fmoc**—9-fluorenylmethoxycarbonyl; **FS-hiPSC-MSCs**—foreskin-derived human induced pluripotent stem cell-derived mesenchymal stem cells; **FTIR**—Fourier-transform infrared spectroscopy; **G-iPSC**—gingival induced pluripotent stem cell; **GA**—gelatin-alginate; **GAHA**—gelatin-alginate-hydroxyapatite; **GAHAp**—gelatin-alginate-hydroxyapatite-plasma rich in growth factors; **GelMA**—gelatin methacryloyl; **GFAP**—glial fibrillary acidic protein; **GFP**—green fluorescent protein; **Gli1**—glioma-associated oncogene homolog 1; **GMSCs**—gingival mesenchymal stem cells; **Gn**—gelatin; **^1^H NMR**—proton nuclear magnetic resonance; **H_2_**—molecular hydrogen; **H_2_S**—hydrogen sulfide; **HA**—hyaluronic acid/hyaluronan; **HA-sECM**—hyaluronic acid-derived extracellular matrix; **hBFP-ADSCs**—human buccal fat pad adipose-derived stem cells; **hBMMSCs**—human bone marrow mesenchymal stem cells; **hBMSCs**—human bone marrow mesenchymal stem cells; **HDAC1**—histone deacetylase 1; **hDPSCs**—human dental pulp stem cells; **HE**—hematoxylin–eosin; **HERS**—Hertwig’s epithelial root sheath; **HGF**—hepatocyte growth factor; **hPDLSCs**—human periodontal ligament stem cells; **hPL**—human platelet lysate; **HRP**—horseradish peroxidase; **hUCMSCs**—human umbilical cord mesenchymal stem cells; **HUVECs**—human umbilical vein endothelial cells; **IGF-1**—insulin-like growth factor-1; **IκB/NF-κB**—inhibitor of κB/nuclear factor kappa B; **IL-1ra**—interleukin-1 receptor antagonist; **IL-4**—interleukin-4; **IL-6**—interleukin-6; **IL-10**—interleukin-10; **iPSC**—induced pluripotent stem cell; **JAK2/STAT3**—Janus kinase 2/signal transducer and activator of transcription 3; **JBMSCs**—jawbone mesenchymal stem cells; **JE**—junctional epithelium; **LacZ**—β-galactosidase reporter gene; **LAMA3**—laminin subunit alpha 3; **Lap**—laponite; **LDH**—lactate dehydrogenase; **LPS**—lipopolysaccharide; **M1**—classically activated macrophage phenotype; **M2**—alternatively activated macrophage phenotype; **MAPK**—mitogen-activated protein kinase; **MEPE**—matrix extracellular phosphoglycoprotein; **micro-CT**—micro-computed tomography; **miR-1246**—microRNA-1246; **MnCO**—manganese carbonyl; **mRNA**—messenger RNA; **MSCs**—mesenchymal stem cells; **MTA**—mineral trioxide aggregate; **MTT**—3-(4,5-dimethylthiazol-2-yl)-2,5-diphenyltetrazolium bromide; **Myf5**—myogenic factor 5; **MyoD**—myogenic differentiation 1; **MyoG**—myogenin; **nanoHA**—nano-hydroxyapatite; **nBGC**—nanoscale bioactive glass ceramic; **NGF**—nerve growth factor; **NRF2**—nuclear factor erythroid 2-related factor 2; **OCN**—osteocalcin; **OPG**—osteoprotegerin; **OpgY**—O-propargyl-tyrosine; **OPN**—osteopontin; **Ost-EV**—osteogenic extracellular vesicle; **PAL**—periodontal attachment level; **PBS**—phosphate-buffered saline; **PCL**—polycaprolactone; **PDGF-BB**—platelet-derived growth factor-BB; **PDL**—periodontal ligament; **PDLSCs**—periodontal ligament stem cells; **PEDOT**—poly(3,4-ethylenedioxythiophene); **PEG**—polyethylene glycol; **PEGDA**—polyethylene glycol diacrylate; **PEGSSDA**—polyethylene glycol diacrylate with an added disulfide bond; **pl-HAM**—polylysine–hyaluronic acid microspheres; **PLA**—polylactic acid; **PLA-HA**—polylactide-hydroxyapatite; **PLGA**—poly(lactic-co-glycolic acid); **PLGA-PEG-PLGA**—poly(lactic-co-glycolic acid)-polyethylene glycol-poly(lactic-co-glycolic acid); **PLLA**—poly-L-lactic acid; **PNIPAAm-g-chitosan**—poly(N-isopropylacrylamide)-grafted chitosan; **POSS**—polyhedral oligomeric silsesquioxane; **ppm**—parts per million; **PRF**—platelet-rich fibrin; **PRGF**—plasma rich in growth factors; **PTHrP**—parathyroid hormone-related protein; **qPCR**—quantitative polymerase chain reaction; **qRT-PCR**—quantitative reverse transcription polymerase chain reaction; **RGD**—arginine-glycine-aspartic acid; **rhBMP-2**—recombinant human bone morphogenetic protein-2; **RNA**—ribonucleic acid; **ROS**—reactive oxygen species; **RT-PCR**—reverse transcription polymerase chain reaction; **RUNX2**—runt-related transcription factor 2; **S-iPSC**—skin induced pluripotent stem cell; **SCAP**—stem cells from the apical papilla; **SCID**—severe combined immunodeficient; **SCX**—scleraxis; **SDF-1**—stromal cell-derived factor 1; **SDF-1α**—stromal cell-derived factor 1 alpha; **SEM**—scanning electron microscopy; **SHED**—stem cells from human exfoliated deciduous teeth; **Shh**—Sonic hedgehog; **SilMA**—silk fibroin methacryloyl; **SOST**—sclerostin; **SOX9**—SRY-box transcription factor 9; **SP7**—Sp7 transcription factor; **STRO-1**—stromal cell antigen-1; **TDN**—tetrahedral DNA nanostructure; **TET2**—ten-eleven translocation methylcytosine dioxygenase 2; **TGF-β1**—transforming growth factor beta 1; **TGF-β3**—transforming growth factor beta 3; **TMJ**—temporomandibular joint; **TMJOA**—temporomandibular joint osteoarthritis; **TNF-α**—tumor necrosis factor-alpha; **TNMD**—tenomodulin; **TPP**—tripolyphosphate; **TRAP**—tartrate-resistant acid phosphatase; **UPy**—ureidopyrimidinone; **UV**—ultraviolet; **VEGF**—vascular endothelial growth factor; **VitD3**—vitamin D3; ***w*/*v***—weight/volume; **WST-1**—water-soluble tetrazolium-1; **wt%**—weight percent; ***wt*/*wt***—weight/weight; **YAP/TAZ**—Yes-associated protein/transcriptional coactivator with PDZ-binding motif; **ZIF-8**—zeolitic imidazolate framework-8; **ΔCAL**—change in clinical attachment level; **ΔGR**—change in gingival recession; **ΔPD**—change in probing depth.
